# Tracking the Photomineralization Mechanism in Irradiated Lab-Generated and Field-Collected Brown Carbon Samples and Its Effect on Cloud Condensation Nuclei Abilities

**DOI:** 10.1021/acsenvironau.2c00055

**Published:** 2023-03-17

**Authors:** Silvan Müller, Chiara Giorio, Nadine Borduas-Dedekind

**Affiliations:** † Department of Environmental Systems Science, ETH Zurich, Zurich 8092, Switzerland; ‡ Yusuf Hamied Department of Chemistry, University of Cambridge, Cambridge CB2 1EW, United Kingdom; ¶ Department of Chemical Sciences, University of Padova, Padova 35131, Italy; § Department of Chemistry, University of British Columbia, Vancouver V6T 1Z1, Canada

**Keywords:** brown carbon, photomineralization, photochemistry, firewood smoke, cloud condensation nuclei

## Abstract

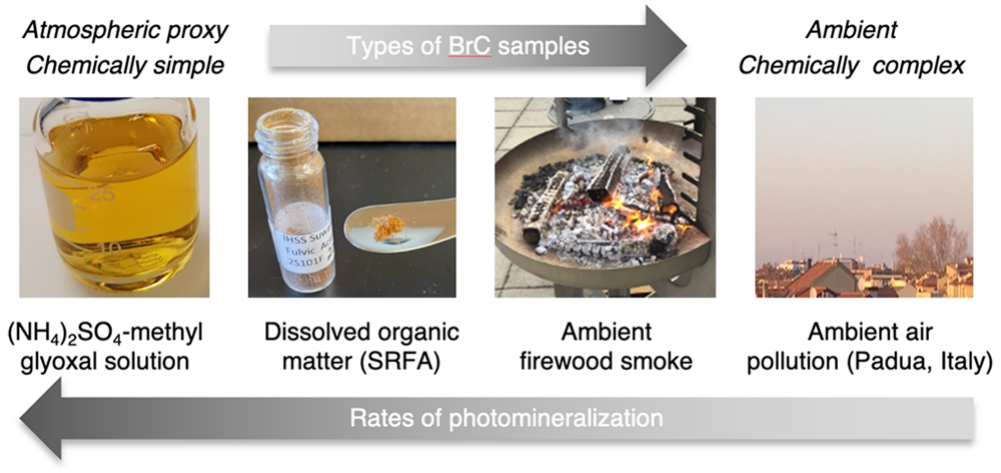

Organic aerosols affect the planet’s radiative balance by absorbing and scattering light as well as by activating cloud droplets. These organic aerosols contain chromophores, termed brown carbon (BrC), and can undergo indirect photochemistry, affecting their ability to act as cloud condensation nuclei (CCN). Here, we investigated the effect of photochemical aging by tracking the conversion of organic carbon into inorganic carbon, termed the photomineralization mechanism, and its effect on the CCN abilities in four different types of BrC samples: (1) laboratory-generated (NH_4_)_2_SO_4_-methylglyoxal solutions, (2) dissolved organic matter isolate from Suwannee River fulvic acid (SRFA), (3) ambient firewood smoke aerosols, and (4) ambient urban wintertime particulate matter in Padua, Italy. Photomineralization occurred in all BrC samples albeit at different rates, evidenced by photobleaching and by loss of organic carbon up to 23% over a simulated 17.6 h of sunlight exposure. These losses were correlated with the production of CO up to 4% and of CO_2_ up to 54% of the initial organic carbon mass, monitored by gas chromatography. Photoproducts of formic, acetic, oxalic and pyruvic acids were also produced during irradiation of the BrC solutions, but at different yields depending on the sample. Despite these chemical changes, CCN abilities did not change substantially for the BrC samples. In fact, the CCN abilities were dictated by the salt content of the BrC solution, trumping a photomineralization effect on the CCN abilities for the hygroscopic BrC samples. Solutions of (NH_4_)_2_SO_4_-methylglyoxal, SRFA, firewood smoke, and ambient Padua samples had hygroscopicity parameters κ of 0.6, 0.1, 0.3, and 0.6, respectively. As expected, the SRFA solution with a κ of 0.1 was most impacted by the photomineralization mechanism. Overall, our results suggest that the photomineralization mechanism is expected in all BrC samples and can drive changes in the optical properties and chemical composition of aging organic aerosols.

## Introduction

Organic aerosols are ubiquitous,^1^ yet their impact on climate and health can be difficult to predict because of their complex location-specific composition, size and morphology. During their atmospheric lifetime, organic aerosols containing visible-light absorbing species, termed brown carbon (BrC), chemically evolve through partitioning and photochemical aging.^2^ This aging process modifies the functional groups within BrC aerosols which impact the aerosol’s ability to scatter and absorb light as well as activate and nucleate clouds thereby impacting the Earth’s radiative budget.^3^

Sources of atmospheric BrC include primary, such as biomass burning, and secondary, such as particulate matter in high NO_*x*_ and/or in high NH_3_ regimes. Determining the identity of the chromophoric molecules responsible for the absorbance of visible light is ongoing research, but is typically thought to occur due to molecules containing heteroatoms, such as N and S, as well as oligomers of aromatic compounds. These BrC components can produce reactive intermediates within the particle such as hydroxyl radicals, singlet oxygen (^1^O_2_), and excited triplet states of organic matter.^4^ These reactive species further contribute to the oxidation of organic aerosols indirectly. Furthermore, photooxidation and photodegradation tend to fragment organic molecules, ultimately converting the organic carbon to CO and CO_2_. This mechanism is subsequently referred to as photomineralization and has been observed in BrC proxies^5,6^ as well as in dissolved organic matter in aquatic systems.^7,8^ This mechanism requires light and oxygen to proceed. The photomineralization mechanism has been extensively documented in aquatic photochemistry of lakes,^9^ rivers,^10,11^ estuaries,^12−15^ and oceans,^16−18^ and we apply this term to the atmospheric chemistry context.

Atmospheric aging mechanisms change organic aerosol properties such as reactivity, size and chemical composition. The light absorption of organic aerosols containing BrC typically decreases with atmospheric aging in a process known as photobleaching.^2^ The rate of photobleaching differs between different types of BrC compounds, as well as on the solvation of the BrC.^19^ These changes in light absorption likely impact the radiative effect of organic aerosols in the atmosphere, though the magnitude of this impact is unknown.^20^

The changes in aerosol properties due to atmospheric aging are important for aerosols’ cloud condensation nuclei (CCN) ability. Aged organic aerosols tend to be more oxygenated and as a result, have more polar and hydrophilic functional groups.^21^ This change increases organic aerosols’ hygroscopicity and consequently increases their propensity to act as CCN, as observed in both field and laboratory studies.^1,22^ However, the source of BrC matters; Mukherjee et al. tested a variety of BrC combustion samples and found that wood, despite having an O/C ratio of 0.4, had a hygroscopicity value κ of less than 0.1.^23^

Oxidative processing increases the hygroscopicity of organic aerosols, but the magnitude of this effect and the resulting influence on atmospheric CCN concentrations is uncertain. To address this issue, Borduas-Dedekind et al. exposed several types of dissolved organic matter (DOM) from surface waters to UVB radiation for up to 25 h, equivalent to 55 h of sunlight.^5^ Subsequent analysis in both CCN and ice nuclei (IN) experiments showed an increase in hygroscopicity up to a factor of 2.5, as well as a decrease in IN activity. These changes in CCN and IN abilities were correlated to a loss of organic carbon with a simultaneous production of CO, CO_2_ during irradiation, indicative of photomineralization.^5^ In this study, we investigate the effect of photochemistry in changing the physicochemical properties of four different BrC samples: ammonium sulfate ((NH_4_)_2_SO_4_)-methylglyoxal solutions, DOM, field-collected biomass burning, and polluted urban particulate matter. Aerosols from firewood smoke were chosen to represent samples typical of biomass burning organic aerosols.

Laboratory-generated BrC, such as (NH_4_)_2_SO_4_-methylglyoxal, are chemically simple and allow a direct comparison to other studies working with the same material (e.g., Wong et al.^20^). To mimic naturally occurring organic matter, DOM standards from the International Humic Substance Society (IHSS) are good proxies, but include additional isolation and extraction steps to generate reproducible samples. However, these lab-generated and lab-altered organic matter samples do not account for the full complexity and representation in chemical composition typically observed in atmospheric aerosols.^24^ To obtain aerosol samples with a higher atmospheric relevance, organic aerosols were collected at the source of firewood smoke, representing fresh biomass burning organic aerosols. Finally, aerosols collected in wintertime Padua, Italy represent real samples of airborne particulate matter from a high pollution urban environment. Padua is located in the Po Valley, a densely populated region in northern Italy which is known to be one of the most polluted areas in Europe.^25^ This work builds upon the results presented by Borduas-Dedekind et al.^5^ by evaluating the photomineralization mechanism within a broader and representative range of BrC samples beyond DOM from surface. The range of investigated BrC samples in this study include artificially generated organic aerosols as well as ambient aerosol extracts in order to draw atmospherically relevant conclusions.

## Materials and Methods

### Sample Description, Collection, and Storage

#### BrC Solution made from (NH_4_)_2_SO_4_ and Methylglyoxal

Aerosols generated from the aqueous reaction of (NH_4_)_2_SO_4_ ((NH_4_)_2_SO_4_) with methylglyoxal are a common proxy for BrC in laboratory studies.^20,26−28^ The solution was prepared according to the procedure described by Wong et al.^20^ First, 0.65 mL of methylglyoxal (40% in water, Sigma-Aldrich, USA) was added to 25 mL of a 1.5 M (NH_4_)_2_SO_4_ solution (Fluka, purity ≥ 99%), prepared using nanopure water (resistivity 18.2 MΩ cm; Milli-Q IQ 7000, Merck, Germany). The final concentration of methylglyoxal was 0.17 M, translating to an organic-to-inorganic ratio of 11.3% by molar concentration, or 6.2% by mass. After mixing, the solution was sealed in an amber bottle which had been prewashed with nanopure water and acetone. The bottle was then wrapped in aluminum foil and left at room temperature in the dark for 2 weeks. During that time, the solution changed from colorless to a dark yellow, indicating the formation of N-containing aromatic compounds (Figure S1). No evaporation by rotavap or by a flow of N_2_ was done on these samples. Three batches of (NH_4_)_2_SO_4_-methylglyoxal solution were generated and aged in the dark for triplicate experiments.

Prior to the photochemical experiments, the solution was diluted to obtain an organic carbon concentration of 20 mg C L^–1^, consistent with the experiments conducted by Borduas-Dedekind et al.^5^ This concentration also corresponds to organic carbon concentrations observed in cloudwater in wildfire-influenced clouds.^29^ In addition, this concentration allows for the chromophoric organic matter to be diluted enough to minimize screening effects during photochemistry.

#### Suwannee River Fulvic Acid (SRFA)

Suwannee River fulvic acid standard (SRFA) (2S101F, International Humic Substance Society (IHSS), USA) was dissolved in nanopure water at a concentration of 20 mg C L^–1^, based on the reported carbon content of 52% by IHSS.^30^ The percentage of inorganic content in this sample is reported by IHSS to be low at 0.58%.

#### Ambient Firewood Smoke

Ambient firewood smoke was collected with a Coriolis μ air sampler (Bertin Technologies, France) (see photo of firewood setup in Figure S2a).^31,32^ During sampling, a vortex is generated inside the sampling cone, allowing the particles to settle and for soluble material to dissolve within the collection liquid of nanopure water. The instrument has been reported to sample larger particles more efficiently than smaller particles.^31,32^ In a study with pollen collection in France, a physical sampling efficiency of 50% was reported for particles 1 μm in diameter, while larger particles (10 μm) were sampled with an efficiency of 92%.^31^ The collection efficiency of soluble material is unknown, but in our sampling campaign, we collected enough material for subsequent experiments.

Prior to sampling, the sampling cones, the air inlet and the air flowing cane were autoclaved (Blanc-Labo, LTA 2×3×4) at 121 °C for 20 min to ensure sterile conditions. Then, 15 mL of the collection liquid, nanopure water, was added to a sampling cone, which was then attached to the air inlet of the instrument (Figure S2a). During an aerosol collection experiment, the air intake flow rate was set to 300 L min^–1^.

Two sets of wood burning experiments were conducted: a wintertime set in November and December 2019 and a summertime set in May, June, July, and August 2020 (Table S1). All experiments were conducted using birch wood logs in a round brazier bowl (Figure S2a). The fire was lighted with matches and facilitated by fire-starters and newspaper. Throughout the duration of the fire, logs were continuously added to ensure a steady supply of smoke. The emitted smoke was sampled by the Coriolis μ air sampler and the device was placed 2 m downwind of the fire (Figure S2a). After 20–30 min of sampling, the cones were replaced with the next cone due to the loss of liquid over time resulting from evaporation. Total collection times ranged between 80 and 240 min (Table S1).

For each firewood experiment, the liquid samples were combined and vacuum-filtered through a 0.2 μm cellulose acetate filter, which was precleaned by filtering 300 mL of nanopure water. This filtration step removed the larger suspended particles including ash. Total organic carbon (TOC) measurements of the solution ranged between 46.8 and 133.9 mg C L^–1^ (Table S1); a range likely due to wind speed and wind direction during the specific sampling day. Based on the TOC measurement, the solutions were diluted down to 20 mg C L^–1^ with nanopure water for the UVB irradiation experiments. The samples were stored in the dark at 4 °C.

Control experiments were conducted where nanopure water was filled into a Coriolis μ sampling cone and filtered according to the same procedure as the firewood smoke samples. These water controls were subsequently used throughout our analyses as blanks and negative controls.

#### Ambient Aerosols in Padua, Italy

Urban wintertime particulate matter (PM) was collected between January 13–17, 2020 at the Department of Chemical Sciences at the University of Padua, in Padua, Italy, located in the polluted Po Valley.^33^ These measurements were taken as part of the PhotOchemIStry, oxidative pOteNtial and tOxicity of Urban aeroSol (POISONOUS) sampling campaign. The site (urban background) is an ideal location for sampling ambient aerosols typical of a polluted urban environment. In the Po Valley, aerosol emissions from traffic, industrial processes and residential heating are known to be the main sources of pollution. However, the morphology of the Po valley also plays a key role in the region’s poor air quality. The mountains surrounding the Po basin–the Alps in the north, and the Apennine mountains in the south–help create stable meteorological conditions in the valley, associated with low ventilation and a shallow boundary layer. These conditions are especially prevalent in wintertime, when low-altitude temperature inversions are common.^25^ As a consequence, emitted aerosols tend to accumulate close to the surface.^34^ During the January 13–17, 2020 sampling period temperatures ranged from −5 to 12 °C, relative humidity ranged between 62 and 100%, wind was calm (<1 m/s), average irradiance was 55 W/m^2^ (max 353 W/m^2^), and PM_2.5_, O_3_, and NO_*x*_ concentrations ranged between 44 and 86 μg/m^3^, 1 and 42 μg/m^3^, and 36 and 425 μg/m^3^, respectively (data from ARPA Veneto, the Environmental Regional Agency).

The Coriolis μ instrument (see Ambient Firewood Smoke section) was positioned close to a window in a room approximately 30 m above ground level such that the air inlet would collect undiluted ambient air (Figure S2b). A colocated optical particle counter (Grimm 1.108, 15 size bins, from 0.23 to 32 μm) was employed to measure the concentration of ambient particulate matter during the measurement campaign (Figure S3).

Sampling took place during the day between 8 AM and 7 PM, with the exception of one additional sample which was collected between 8 PM and 10 PM (Table S2). For each sample, the Coriolis μ instrument was in operation for a total of 120 min. To correct for the loss of liquid due to evaporation (approximately 3 mL per 10 min interval), the cones were disconnected from the instrument every 30 min and refilled to 15 mL with nanopure water. This step was necessary to optimize the collection efficiency.^31^

Back in the laboratory, the samples obtained in Padua were treated the same as the firewood samples and were vacuum-filtered through a precleaned 0.2 μm cellulose acetate filter. In this step, some of the samples were combined to optimize TOC content (Table S2). Only the Friday (January 17, 2020) sample proved to be sufficiently concentrated in organic material to be used for photochemical experiments at 20 mg C L^–1^ (Table S2).

### Photochemical Experiments

#### Photochemical Reactor

To simulate atmospheric aging by sunlight, samples were exposed to six UVB bulbs in a commercial photoreactor (Rayonet model RPR-100, Southern New England Ultraviolet Co, USA) at 30 °C. The UVB bulbs employed in the photoreactor emit radiation between wavelengths 280 and 400 nm, with a peak at 313 nm (Figure S5). Borosilicate test tubes (10 mL in size) were filled with 9 mL of sample, capped with a cork stopper and placed on a rotating turn-table at the center of the photoreactor. At each time point, test tubes were removed and replaced by test tubes containing nanopure water only, to ensure constant light path throughout the irradiation experiments. They were then transferred to a precleaned amber vial and stored in the fridge at 4 °C. The cleaning procedure for the amber vials involved washing with water as well as acetone, followed by drying for at least 1 h in an oven at 120 °C. Time point zero was also treated the same way. Furthermore, previous control experiments with vials wrapped in aluminum foil showed that the change in temperature alone does not induce any chemical reactions related to photomineralization in DOM samples.^5^

#### Chemical Actinometry

A conversion of the photon flux in this photoreactor setup to equivalent hours of sunlight has previously been established by Borduas-Dedekind et al.^5^ Briefly, a chemical actinometer, pyridine/*p*-nitroanisole,^35^ was irradiated for 5 h in the same photoreactor using the same six UVB bulbs. The observed degradation of pyridine/*p*-nitroanisole over time was determined with high-performance liquid chromatography, and yielded an integrated irradiance (280–400 nm) of 64 ± 4 J m^–2^ s^–1^. This result was compared to the solar spectrum of an atmospheric reference, obtained from the Simple Model of the Atmospheric Radiative Transfer of Sunshine (SMARTS, NREL). The integrated irradiance for a solar spectrum typical for midlatitudes (45°) in summer suggested a conversion factor of 2.2 compared to the photoreactor setup. Thus, UVB irradiation in the photoreactor can be converted to an equivalent irradiation time in natural sunlight. A photoreactor irradiation time of 8 h is equivalent to 17.6 h of sunlight exposure. However, this estimate does not take into account cloud cover or changes in zenith angles and consequently represents an upper bound. Overall, the photochemistry experiments described in this section equate to 1–2 days in the atmosphere, assuming 12 h of light per day in clear conditions.^5^

### Analytical Chemistry Instruments

#### Total Organic Carbon Analyzer

A total organic carbon (TOC) analyzer (TOC-L CSH, Shimadzu, Japan) was used to quantify the nonpurgeable organic carbon (NPOC) in the aqueous samples. The measurement method involved the injections of 50 μL of sample, a purge time of 1.5 min and a gas flow of 80 mL min^–1^. The organic carbon is then oxidized to CO_2_ over a heated catalyst column and is then detected by infrared absorbance. For each sample, three to four measurements were made depending on the standard deviation of the first three measurements. The TOC analyzer was calibrated daily between 1 and 20 mg C L^–1^, using a potassium terephthalate TOC standard (Sigma-Aldrich). For the TOC measurements of (NH_4_)_2_SO_4_-methylglyoxal, an imidazole solution in the same concentration range was prepared to better mimic the organic compounds in the sample compared to the TOC standard. The TOC values reported are likely a lower limit as we observed insufficient recovery of TOC concentrations with known concentrations of lignin, a macromolecule relevant to biomass burning aerosols (see the Supporting Information (SI) for discussion and Figure S4).

#### UV–Visible Spectroscopy

The changing absorbance of the BrC samples during irradiation was monitored by a UV–visible (UV/vis) spectrophotometer (Cary 100, Varian Inc., USA) over the range of 200–800 nm. The absorbance of the samples is also reported as the mass absorption coefficient (MAC). The MAC is calculated according to eq 1:^27^
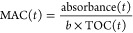
1where *b* is the path length (1 cm) and where TOC is in g cm^–3^. For each time point during UVB irradiation, the MAC value of the sample was calculated as the absorbance divided by the TOC concentration at that time point. We also use the MAC_300 nm_ value to represent TOC-normalized changes in absorbance at wavelength 300 nm; a wavelength arbitrarily chosen as a reference point to track photobleaching.

#### pH and Conductivity

The pH of the samples was measured with an electronic pH meter (691 pH meter; Metrohm AG, Switzerland), after calibration with pH 4 and pH 7 buffer solutions. To measure conductivity, a portable conductivity meter (LAQUA twin model EC-33, Horiba Scientific, Japan) was used. Calibration of the instrument was done using a 1413 μS cm^–1^ standard solution.

#### Ion Chromatography

Acetic, formic, oxalic, and pyruvic acids are low molecular weight organic compounds formed during the photodegradation of DOM^5^ and were quantified using an ion chromatograph (Dionex DX-320, Thermo Fisher Scientific, USA) equipped with a Dionex Ion-Pac AG11-HC RFIC 4 mm column and guard column, a 4 mm suppressor of 40 mA (Dionex Aers 500), and a conductivity detector. An EG40 eluent generator was used to generate a KOH concentration gradient from 1 mM (constant from 0 to 14 min) to 35 mM (linearly increasing from 14 to 31 min). Injection volume and flow rate were 250 μL and 1.5 mL min^–1^, respectively. To calibrate the concentrations of the organic acids, standard solutions of the four organic acids were prepared in nanopure water and repeated at four different intervals during the experimental period of 9 months (Figure S10). The most recent calibration was applied to the measurement. The retention times of the organic acids in this calibration were 8.0, 10.7, 13.7, and 26.3 min for acetic, formic, pyruvic, and oxalic acids, respectively. However, these retention times differed for the (NH_4_)_2_SO_4_-methylglyoxal solutions, likely due to interference from the high concentrations of sulfate (4.9 mM). To calibrate for this effect, each of the organic acids was added to a separate solution of (NH_4_)_2_SO_4_-methylglyoxal (50 mM). The corrected retention times in these samples were 6.7 min, 9.2 and 12.2 min for acetic, formic and pyruvic acids, respectively. The retention time of oxalic acid remained unchanged.

#### Gas Chromatography

To quantify CO and CO_2_ production during UVB irradiation, the photochemical experimental setup had to be adapted to prevent the partitioning and thus loss of these gas-phase photoproducts from the BrC solutions, similarly to Borduas-Dedekind et al.^5^ Headspace-free samples were prepared by sealing the borosilicate test tubes with a rubber septa and subsequently irradiated in the same Rayonet photoreactor setup with six UVB bulbs. The number of time points were adapted based on the available sample volume, which was limited for the firewood and Padua BrC samples. After irradiation, samples were transferred by syringe to 20 mL serum vials which had been sealed airtight, flushed with N_2_ (approximately 0.5 bar overpressure) and filled with 0.1 mL of 1 N HCl. The acidic conditions shifts the carbonate equilibrium to CO_2_. Samples were kept at 4 °C and measured within a week of the photochemical experiments.

The CO and CO_2_ concentrations in the static headspace of the serum vials were sampled at room temperature and measured with a gas chromatography system equipped with a methanizer and a flame ionization detector (GC-FID; SRI Instruments, Menlo Park, USA). Separation was done over a 2.7 m HayeSep D column with N_2_ as the carrier gas. The instrument was calibrated for the concentration range of 5 to 100 ppm for CO and 50 to 2000 ppm for CO_2_ using standard gas cylinders (Figure S11). The GC-FID measures the partial pressure of CO and CO_2_ in the headspace (*p*_CO,hs_, *p*_CO_2_,hs_) and the corresponding concentrations of CO and CO_2_ within the solution were calculated using the ideal gas law and Henry’s law. The pressure in the serum vial headspace (*p*_tot,hs_) and the atmospheric pressure (*p*_atm_) were measured with a barometer prior to each measurement, and the sample volume (*V*_aq_) was determined by weighing the vials before and after the injection of the sample. First, the moles of CO and CO_2_ in the gas phase were calculated with eq 2 via the ideal gas law:
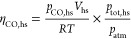
2where *V*_hs_ is the volume of the headspace calculated as the vial volume minus (*V*_aq_), *R* is the ideal gas constant, and *T* is the temperature at the time of measurement. The moles of CO and CO_2_ in the aqueous phase at the equilibrium (*n*_CO,aq_) were then determined via Henry’s law, eq 3:
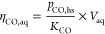
3where *K*_CO_ and *K*_CO_2__ are the temperature-corrected Henry’s constant from Fry et al.^36^ Finally, the initial concentration of CO and CO_2_ in the aqueous phase ([CO]_aq_^0^,[CO_2_]_aq_^0^) can be calculated with eq 4:
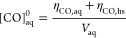
4

### Cloud Condensation Nuclei Experiments

The CCN ability of the aerosol samples was determined using a continuous-flow thermal-gradient cloud condensation nuclei chamber (CCNC) (Droplet Measurement Technologies, USA).^37,38^ The experimental setup employed in this work is identical to the one described and depicted in Borduas-Dedekind et al.^6^ The samples were first aerosolized by a custom-built atomizer, based on the aerosol generator model 3076 by TSI Incorporated (USA). The produced flow of wet aerosols was dried through two diffusion driers containing first, silica gel and subsequently, molecular sieves. A differential mobility analyzer (DMA; TSI, model 3082), equipped with a radioactive Po-210 bipolar diffusion charger, was then used to select a monodisperse flow of aerosols with diameters between 20 and 100 nm. Then, the flow was split between the CCNC at a flow rate of 0.5 L min^–1^, and a condensation particle counter (CPC; TSI, model 3772) at a flow rate of 1.0 L min^–1^. The size selected and dried aerosols then entered the CCNC growth column, where they were exposed to supersaturated conditions and a continuous temperature gradient along the vertical stream direction. The supersaturation conditions used in this work ranged between 0.3% and 0.6% depending on the BrC samples (Figure S17a). These conditions allowed the droplets to grow continuously throughout the instrument.^37^ At the outlet of the growth column, the optical particle counter (OPC) detects particles larger than 0.75 μm, which are counted as cloud droplets.

By comparing the time-synchronized data provided by the particle counter and the CCNC, the fraction of particles that acted as CCN, labeled as the CCN fraction, was calculated for a given particle diameter and supersaturation, and repeated to obtain the sample’s CCN activation curve (Figure S17). These curves were produced by keeping the supersaturation constant while changing the particle size, since the diameters can be scanned more quickly than the supersaturation conditions allowing for more efficient analysis. The CCN activation curve was then generated by plotting the CCN fraction of each diameter on a graph and fitting these points with a sigmoidal curve (Figure S17). See the SI for additional details on the CCNC calibration (Figure S16) and control and reproducibility experiments.

The CCN activation curves enable the calculation of the hygroscopicity parameter κ, using eqs 1 and 2:^39^
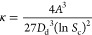
5
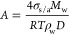
6where *D*_d_ is the critical diameter, i.e., the diameter at which 50% of the particles activated into droplets for a given supersaturation *S*_c_; σ_s/a_, *M*_w_, and ρ_w_ are the surface tension (0.072 J m^–2^), molecular weight (18 g mol^–1^), and density (10^6^ g m^–3^) of water, respectively; *R* is the universal gas constant (8.314 J K^–1^ mol^–1^), and *T* is the temperature of the flow going into the CCNC (25 °C or 298 K).

The CCN ability of the sample is ultimately determined by comparing the number of detected cloud droplets formed in the CCNC with the total particle number detected by the CPC. One of the caveats related to this setup is the possibility of chemical transformations of the sample between the initial aerosolization and the final analysis in the CCNC. For example, the drying process may impact the pH of the sample and thus alter its chemistry. However, these changes would be consistent across samples and thus a change in CCN activity can be related to a change in the chemical composition of the atomized BrC solution sample.

Of note, we attempted to measure the ice nucleation (IN) activity of the BrC samples using our home-built Drop Freezing Ice Nuclei Counter (FINC).^40^ However, the IN activity of the BrC solutions was at the limit of detection of the handling blank control and thus the data was inconclusive. We include this information as a warning of high background IN activity when operating with glassware, different types of water sources and ambient samples.^40−42^

## Results and Discussion

The composition of atmospheric aerosols is temporally and spatially variable and depends on local and regional sources. To reflect this heterogeneity, experiments in this study were conducted on BrC of four different sources: (1) (NH_4_)_2_SO_4_-methylglyoxal solutions, (2) SRFA solutions, (3) firewood aerosol extracts, and (4) wintertime polluted ambient air in Padua, Italy. Solutions were prepared and/or diluted with a target organic carbon concentration representative of cloudwater concentrations around 20 mg C^–1^ L, equivalent to 1670 μM of organic carbon.^29^

### Photobleaching

**Figure 1:**
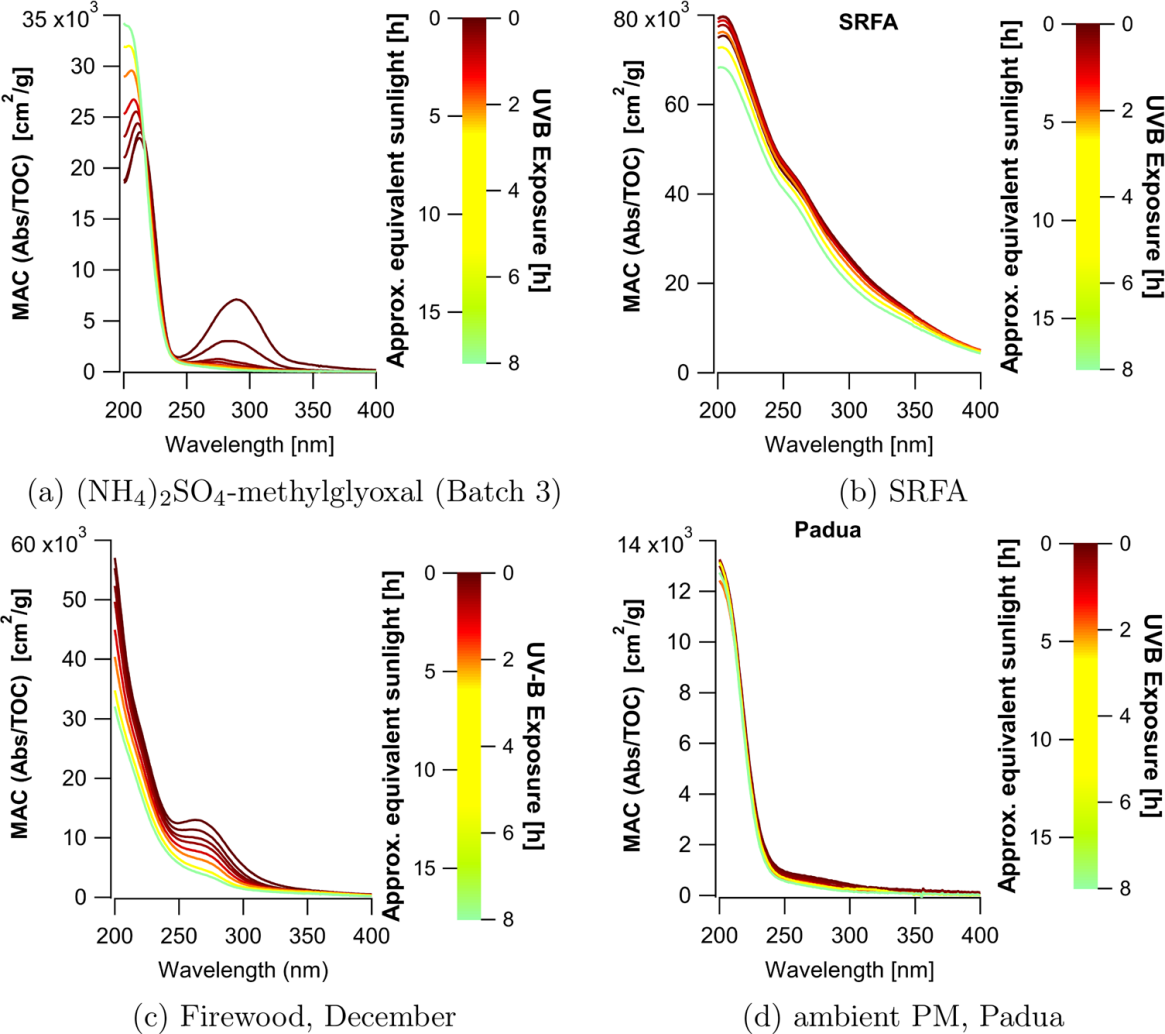
Spectra of mass absorption coefficient (MAC) values (in cm^2^/g) between 200 and 400 nm for (a) one batch of (NH_4_)_2_SO_4_-methylglyoxal, (b) SRFA solutions, (c) firewood smoke December sample, and (d) Padua sample. Changes in absorbance due to UVB irradiation are indicated by a color scale. Note that differences between the spectra below ≈230 nm, for example, in (a), are not relevant for photomineralization since the employed UVB bulbs during photochemical treatment do not emit radiation at these wavelengths. Note also that the *y*-axes have different scales for each panel to highlight the photobleaching as best as possible.

All BrC samples exhibited photobleaching, albeit to varying degrees (Figure 1). Most of the chromophores formed from the reaction of (NH_4_)_2_SO_4_ with methylglyoxal are organic N-containing compounds, some of which likely contain aromatic rings such as imidazoles.^28,43−47^ The absorption spectra of the solutions of (NH_4_)_2_SO_4_-methylglyoxal studied here show an absorption peak at 290 nm, as observed in other studies with this type of laboratory BrC (Figure 1).^48^ The intensity of this peak quickly decreases within the first hour of UVB exposure, indicating a rapid rate of photobleaching of these chromophores. Indeed, all three batches of (NH_4_)_2_SO_4_-methylglyoxal showed an exponential decay in MAC at 300 nm (Figure S9, top panel), evidence of highly labile chromophores, likely N-containing oligomers.^47,49^ An exponential fit through the data yields the equation:
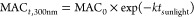
7which expresses the decrease in MAC at 300 nm as a function of hours of sunlight exposure (*t*_sunlight_),^27^ from an initial value MAC_0_ of 5.51 × 10^3^ cm^2^ g^–1^. The first-order rate constant, *k*, is 4.02 h^–1^ for (NH_4_)_2_SO_4_-methylglyoxal and 82 h^–1^ for firewood smoke against photobleaching for the chromophores absorbing at 300 nm (Figure S9). A similar exponential decay in the absorbance of (NH_4_)_2_SO_4_-methylglyoxal in response to broadband UV irradiation has been reported by Aiona et al.^48^ Our results support the overall conclusion that (NH_4_)_2_SO_4_-methylglyoxal BrC is expected to photobleach faster than the other BrC samples. This rate difference impacts the extrapolation of lab measurements using proxies such as (NH_4_)_2_SO_4_-methylglyoxal to real atmospheric photobleaching.

SRFA solutions had the highest MAC values in the visible range and experienced some photobleaching as expected from others’ observations (Figure 1b).^5,50^ Extended irradiation experiments to 25 h showed no further decreases in MAC values (Figure S8).

The absorbance spectra of the nonirradiated firewood smoke aerosol samples exhibit a local maximum in the MAC spectra at ≈270 nm (Figure 1c). The shape of the absorption spectra, and the changes observed, show similarities to photobleaching of lignin.^51^ Since lignin is present in all terrestrial plants, its chromophores likely account for some of the absorbance in the firewood smoke samples. Despite the higher organic carbon concentration in the November sample, the MAC_300nm_ values and their change over time were similar in both samples. The MAC_300nm_ value decreased quickly by 50% within 1 h of UVB exposure, or approximately 2 h of equivalent sunlight. This initial rapid decay was followed by a slower rate of photobleaching throughout the rest of the experiment (Figure S9 bottom panel); photobleaching was considerably slower than the (NH_4_)_2_SO_4_ methylglyoxal solutions (Figures 1c and S8). Aerosols from firewood smoke thus appeared to contain a persistent population of chromophores which continued to degrade throughout the entire duration of UVB exposure, in line with previous observations by Wong et al.^20^ A linear fit of the data points from 1 to 8 h of UVB irradiation yielded a slope of −82 h_sunlight_^–1^. With a MAC_300nm_ of 2.6 × 10^3^ g cm^2^ for both firewood smoke samples at the start of this linear phase, it would take 16 h of sunlight for the absorbance to be decreased by half.

The aerosols collected in Padua were characterized by low absorbance in the UVA and UVB range. At 300 nm, the MAC of the nonirradiated sample was an order of magnitude lower than for the firewood smoke aerosols (Figure 1c,d). Lower absorbance is likely due to lower concentrations of chromophores and therefore less overlap with the irradiance of the photoreactor. Nonetheless, photobleaching was still observed, as the MAC decreased by 50% over 8 h of UVB exposure (Figure 1d). We observed low absorbance of this sample in comparison to the firewood smoke samples, likely due to prior photooxidation of BrC originating from residential heating in wintertime Padua.^25^ Note that there was a wood burning restriction in place in Padua from October 2019 to March 2020. Nonetheless, combustion generated aerosols from traffic emissions are also an important source of BrC and black carbon locally. These soot aerosols are highly absorbing,^52^ but insoluble, and would have been removed during the filtration step. In a study on the optical properties of urban aerosols in Bologna in the Po Valley, Costabile et al. found that the absorbance of the aerosols strongly depended on the ratio of organic carbon to black carbon.^53^ Aerosols dominated by black carbon, and consisting of aromatic carbon moieties^52^ were observed to be much more absorbing than aerosols containing a higher fraction of organic carbon.

### Organic Carbon Concentrations

**Figure 2:**
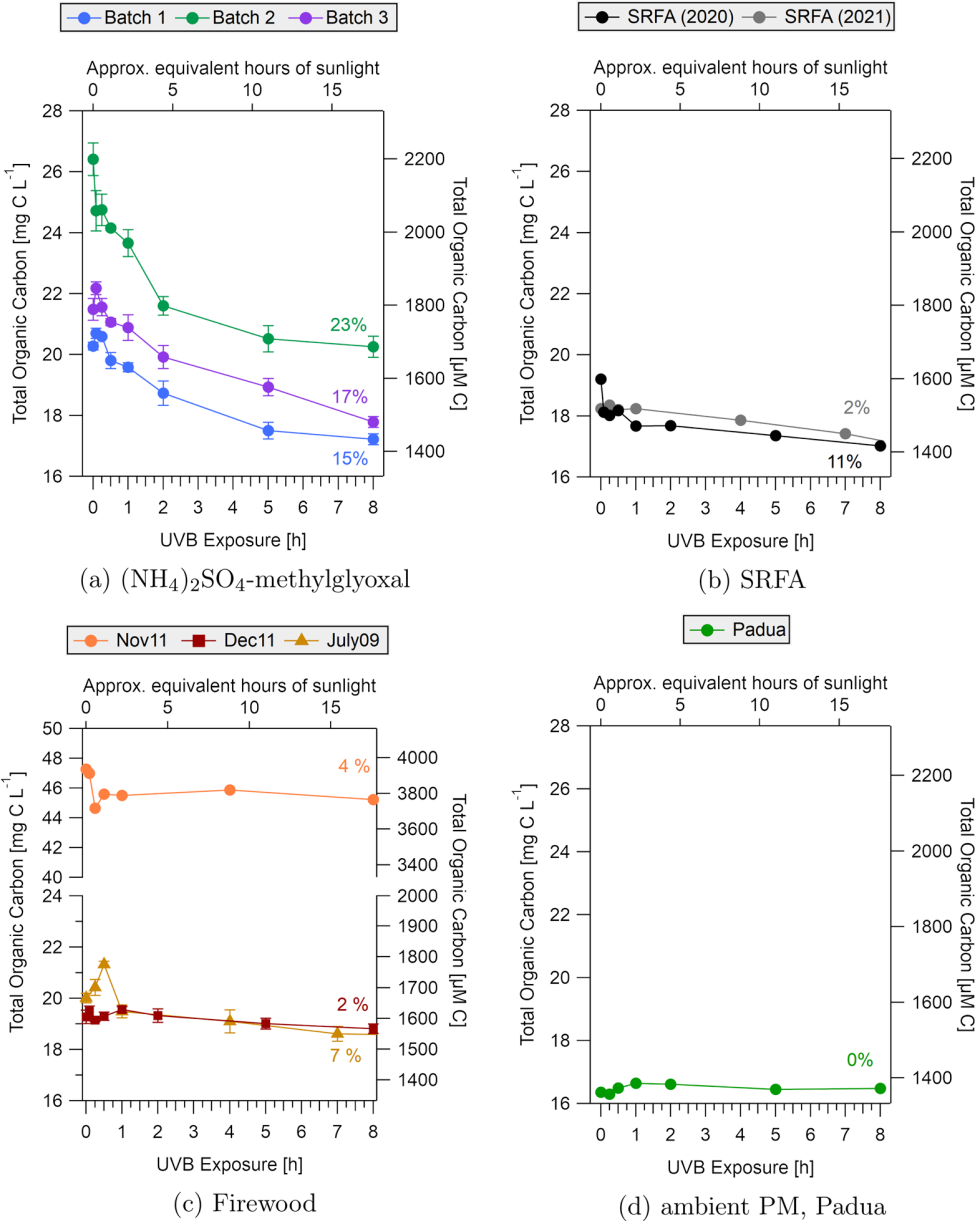
Total organic carbon (TOC) concentration (a) in (NH_4_)_2_SO_4_-methylglyoxal solutions, (b) in SRFA, (c) in the firewood samples (note the different axes), and (d) in the Padua sample as a function of UVB irradiation (bottom *x*-axis) and as a function of equivalent hours of sunlight (top *x*-axis). Concentrations are given in mg C L^–1^ as well as in μM of C, and for each sample, the overall loss of organic carbon is given as a percentage. Error bars indicate the standard deviation of triplicate measurements made by the TOC analyzer.

Organic carbon concentrations measured by a Shimadzu total organic carbon (TOC) analyzer decreased as a function of UVB exposure up to 23% after an equivalent sunlight exposure of 17.6 h, except for the ambient Padua BrC sample (Figure 2).

The reaction products formed from the reaction of (NH_4_)_2_SO_4_ and methylglyoxal include N-containing compounds such as imidazoles,^49,54^ though high-molecular-weight oligomeric species have also been observed.^55,56^ In the three batches of (NH_4_)_2_SO_4_-methylglyoxal, between 15 and 23% of the organic carbon initially present was mineralized after 8 h of UVB irradiation (Figure 2a). Note that the slight differences in TOC concentrations of the initial solutions likely stem from difficulties in measuring methylglyoxal concentrations accurately as it is a volatile compound. Furthermore, slight deviations in organic carbon concentrations could arise from ammonium salts, which are known to affect the solubility of methylglyoxal and other organics.^57^

The SRFA solutions exhibited loss of organic carbon as expected from Borduas-Dedekind et al.^5^ (Figures 2b and S6). Most of the decay occurs at the earliest time points, concurrent with a faster photobleaching rate in SRFA solutions.

In the ambient aerosol samples, photomineralization was less efficient than that for (NH_4_)_2_SO_4_-methylglyoxal. Organic carbon content decreased by 2.0 mg C L^–1^ over 8 h for the firewood smoke sample obtained in November, equivalent to a loss of 4% of the initial TOC content (Figure 2c). Extended irradiation time led to decreases in TOC up to 7% but with some variability (Figure S7). For the *December* sample, a loss of 0.5 mg C L^–1^ was observed, which translates to a loss of 2.4% compared to the nonirradiated sample. Even with samples containing twice as much TOC content as in the *November* sample, the loss of carbon remained proportional. We also discuss in the SI possible issues when measuring the TOC of BrC on a Shimadzu instrument and conclude that our values represent a lower estimate of the extent of carbon loss in our photochemical experiments (see the SI and Figure S4). In a similar experiment, Wong et al.^20^ exposed wood smoke BrC samples to UVA irradiation and reported a loss of 30% of the water-soluble organic carbon after 125 h, and approximately 10% after 10 h. However, an important difference in their experimental setup is the use of an anoxic pyrolysis setup to collect wood smoke aerosols, which suppresses the formation of black carbon. The wood burning aerosols were sampled outdoors at real ambient conditions of biomass burning emissions. As a result, the obtained samples may contain a higher fraction of photorecalcitrant material.

Loss of organic carbon was not observed in the wintertime ambient Padua sample extract, suggesting that exposure to UVB radiation for 8 h oxidized only a minor fraction of organic matter (Figure 2d). The analysis of CO, CO_2_ and organic acid production corroborate the low rate of photomineralization (see Table 1). While the chemical composition of this sample was not quantified, the main sources of primary organic aerosols in the Po Valley are residential heating and traffic emissions.^58^ Organic aerosols from combustion processes may contain substantial amounts of high molecular weight polycyclic aromatic hydrocarbons, which exhibit a wide range of aqueous photodegradation rates.^59^ The low photodegradation rate observed here is likely related to the low rate of absorbance of the organic matter in this sample, thereby limiting the absorbance overlap between the photoreactor and the sample.

**Table 1:** Summary of the Photomineralization Mechanism in Four Irradiated Lab-Generated and Field-Collected BrC Samples over 8 h of Irradiation Equivalent to 17.6 h of Sunlight Exposure^a^

BrC sample	(NH_4_)_2_SO_4_-methylglyoxal	SRFA	firewood	Padua
photobleaching at 300 nm	<100%	<20%	<73%	58%
TOC of BrC	1700–2200 μM	1600 μM	1600 μM	1400 μM
loss of TOC	15–23%	11%	2–4%	0%
production of organic acids	highest	moderate	moderate	lowest
production of CO	<54 μM	9 μM	9 μM	2 μM
production of CO_2_	<1032 μM	315 μM	115 μM	24 μM
rate of photomineralization	fastest	moderate	moderate	slowest

a Despite similar concentrations of TOC, different photoproducts are produced at different rates within the range of BrC samples.

### pH and Conductivity

The pH of the aerosol samples is an important parameter to measure as it dictates acid–base chemistry.^60^ In the atmosphere, aerosol droplets are generally acidic, with lower pH reported for aerosols influenced by anthropogenic emissions.^61^ Conductivity is a measure of the total quantity of ions in the solution, and thus provides an estimate of the abundance of inorganic ions observed in our BrC samples such as sulfate, nitrate, carbonate, halogens and ammonium.^62^

The nonirradiated samples of (NH_4_)_2_SO_4_-methylglyoxal had lower pH, between 4.26 and 4.55, and high conductivity, between 1277 and 1346 μS cm^–1^ (Figure S14). Most of the changes due to UVB exposure occurred within the first 2 h, including a decrease of the pH down to 3.84 ± 0.01 for all three batches, as well as an increase in conductivity by 40 to 50 μS cm^–1^. Both of these changes can be attributed to the production of organic acids as a source of both acidity and ionic species. The pH and conductivity of SRFA solutions under the same experimental conditions were reported to be 5 and 30 μS cm^–1^ and remained unchanged during irradiation.^5^

For the firewood smoke samples, the initial pH of 4.67 (November) and 4.80 (December) remained unchanged throughout the duration of UVB irradiation (Figure S15). The conductivity was low for both samples between 22 and 29 μS cm^–1^ for the November sample and between 34 and 50 μS cm^–1^ for the December sample. These results indicate a negligible contribution of ionic species to the firewood smoke samples.

The Padua Friday sample, on the other hand, was characterized by similarly low conductivity but a higher pH of 7.4 which decreased to 6.9 over 8 h of UVB irradiation (Figure S15). This higher pH could have resulted from the buffering capacity of ammonia, which was indeed measured by IC to be 3396 ng m^−3^ and observed to be present in the Po Valley atmosphere due to emissions from agriculture.^62^ However, the low conductivity of the Padua Friday sample suggests that few ions were present, and so the buffering capacity could have also originated from the little organic carbon content.

### Photoproducts

#### Production of Organic Acids from Lab-Generated BrC Samples

Acetic acid was present before irradiation in all samples tested (Table S5). In (NH_4_)_2_SO_4_-methylglyoxal solutions, initial concentrations ranged between 320 and 360 μM (Table S5). Yu et al. observed the production of both acetic acid and formic acid from the reaction of 1 M methylglyoxal with 1 M (NH_4_)_2_SO_4_ in solution.^45^ While methylglyoxal concentrations were lower in the (NH_4_)_2_SO_4_-methylglyoxal solutions studied, the initial concentrations of these organic acids can potentially be attributed to their production during the browning process of the solution.

**Figure 3:**
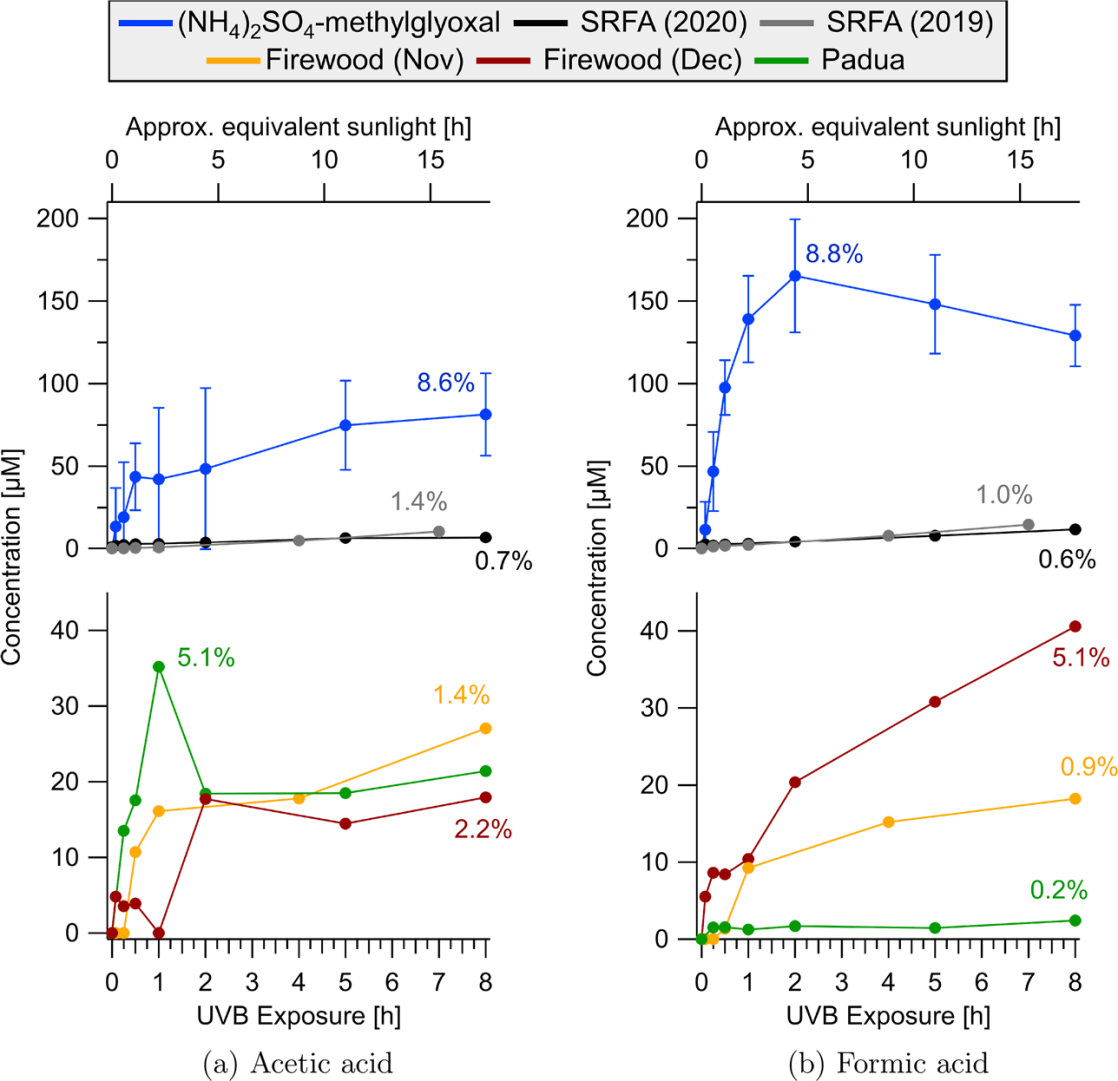
Acetic acid (a) and formic acid (b) concentrations measured by IC as a function of UVB irradiation for the four types of BrC samples. Top panels and bottom panels show results for the lab-generated and field-collected BrC samples, respectively. The (NH_4_)_2_SO_4_-methylglyoxal solution time points were averaged and standard deviations are shown, as we expected this laboratory-generated sample to give reproducible results. Percentages indicate the maximum yield of the acid produced over 8 h from the initial TOC content in Figure 2. Concentrations of organic acids for extended irradiation up to 25 h are shown in Figure S12.

**Figure 4:**
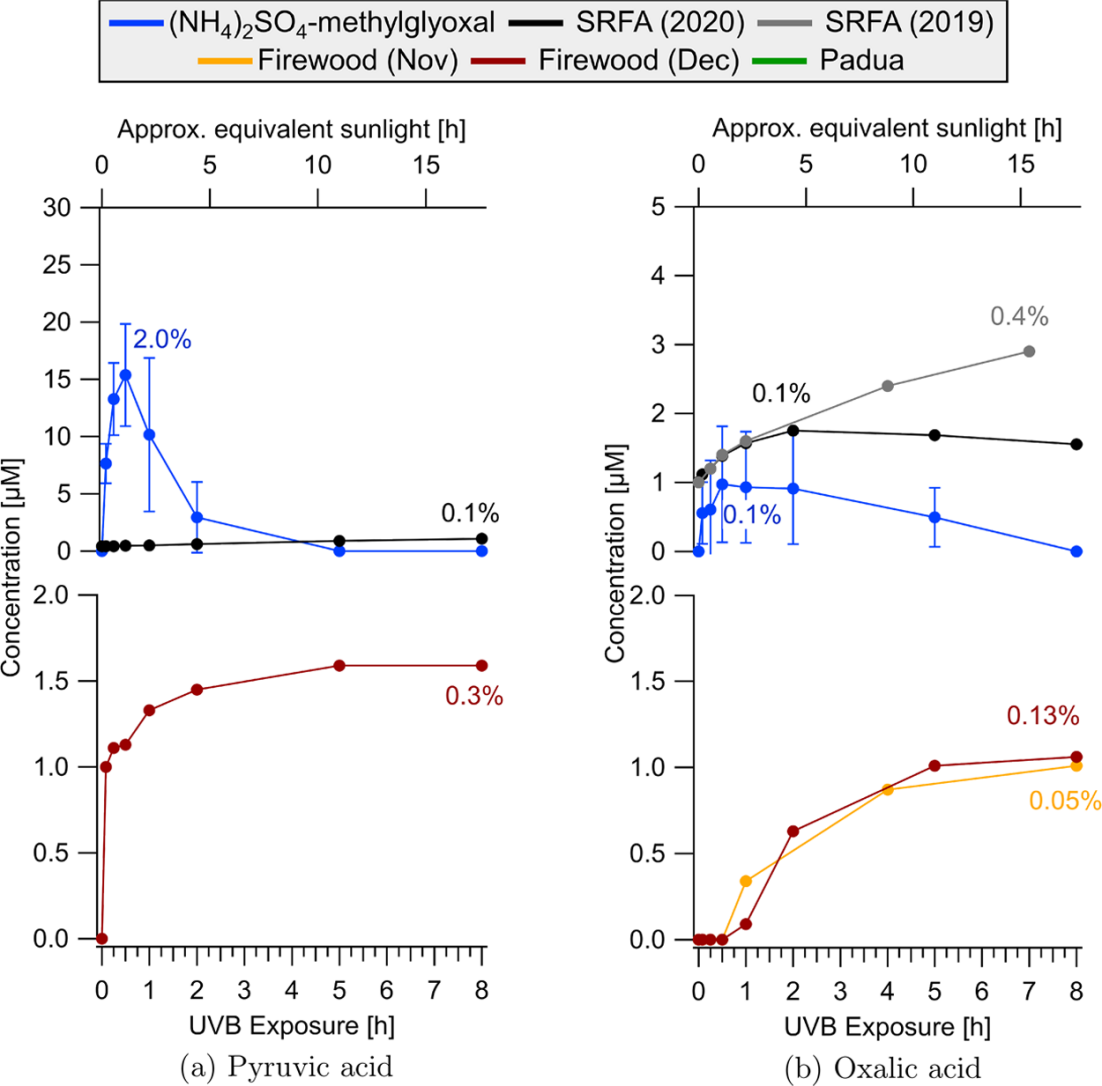
Pyruvic acid (a) and oxalic acid (b) concentrations measured by IC as a function of UVB irradiation for the four types of BrC samples. Top panels and bottom panels show results for the lab-generated and field-collected BrC samples, respectively. The (NH_4_)_2_SO_4_-methylglyoxal solution time points were averaged and standard deviations are shown, as we expected this laboratory-generated sample to give reproducible results. Percentages indicate the maximum yield of the acid produced over 8 h from the initial TOC content in Figure 2. Samples not included did not show acid production above the detection limit. Concentrations of organic acids for extended irradiation up to 25 h are shown in Figure S12.

Acetic acid, formic acid, pyruvic acid and oxalic acid are products formed from the photooxidation of organic matter and represent intermediates in the photomineralization mechanism.^5^ These organic acids were quantified via ion chromatography during UVB irradiation (Figures 3 and 4).

In the (NH_4_)_2_SO_4_-methylglyoxal samples, formic acid increased in concentration from between 30 and 40 μM up to 200 μM in all three batches within the first 2 h of UVB exposure. This large production of formic acid likely contributed to the decrease in pH observed. Production of pyruvic acid appeared to be rapid, with maximum concentrations between 10 and 20 μM after 0.5 to 1 h of UVB irradiation, followed by a decrease to below 5 μM. Oxalic acid was not detected in any of the nonirradiated BrC solutions, and increased to 2–4 μM during UVB exposure. Oxalic acid is therefore a photogenerated product and a likely tracer for photochemistry.

#### Growth and Decay Box Model of Formic and Pyruvic Acids

Furthermore, the time series of formic acid and pyruvic acid exhibit typical features of growth-and-decay curves, and can thus be fitted to a simple kinetic box model (Table S4). The box model equation expresses the concentration of formic acid over time through its production from the photooxidation of BrC and its removal through its reactive sinks:
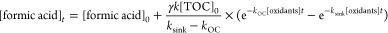
8where [formic acid]_0_ is the concentration of formic acid in the nonirradiated sample; [TOC] is the concentration of the available organic carbon for formic acid production, and [oxidants] the concentration of all possible oxidants for that process; *k*_*OC*_ and γ are the rate constant and yield, respectively, for the production of formic acid from photooxidation of organic compounds. The rate constant *k*_sink_, in combination with the concentration [oxidants], represents the sum of all sink processes by which formic acid was depleted (values are listed in Table S4).

In this parametrization, [TOC] is assumed to be equal to the total initial organic carbon concentration in the sample, 1600 μM or approximately 20 mg C L^–1^, representing an upper bound estimate. The average initial concentration of formic acid ([formic acid]_0_) of all three batches of (NH_4_)_2_SO_4_-methylglyoxal was 39 × 10^–6^ M. For *k*_OC_, a value of 5.6 × 10^9^ M^–1^ s^–1^ was chosen based on the representative rate constant of organic molecules with hydroxyl radicals.^63^ The associated yield γ is best fitted with 14% (Table S4). The sum of the sink processes was represented via the parameters *k*_sink_ and [oxidants]. A rate constant *k*_sink_ of 4 × 10^8^ M^–1^ s^–1^ produced the best fit to the data, combined with a [oxidants] concentration of 5 × 10^–14^ M which is on the same order of magnitude as the steady-state concentration of singlet oxygen (^1^O_2_) in irradiated solutions (Table S4).^64,65^

The kinetic box model for pyruvic acid retained the same values for [TOC], *k*_OC_ and [oxidants]. The initial concentration in (NH_4_)_2_SO_4_-methylglyoxal solutions was on average 2.1 × 10^–6^ M, and a photooxidation yield of γ of 0.04% best fitted the observed production. The sink term appeared to be more dominant than in formic acid, as it dominates over the production term from 1 h of UVB irradiation onward. As a consequence, a high rate constant *k*_*sink*_ of 10^10^ M^–1^ s^–1^ was needed to fit the data, equivalent to the limit for diffusion limited reactions.^63^ A summary of all parameters used in the kinetic models is provided in the SI.

The kinetic models of both formic acid and pyruvic acid indicate that sink processes other than the reaction with hydroxyl radicals dominated the removal of these compounds. Multiplying the steady-state concentration of hydroxyl radicals determined for irradiated solutions (3.6 × 10^–17^ M;^64^) with the reaction rate constant for formic acid (2.4 × 10^9^ M^–1^ s^–1^;^66^) yields a rate of 8.6 × 10^–8^ s^–1^, 3 orders of magnitude lower than the combined sink term in the kinetic model (*k*_sink_ × [oxidants] = 2 × 10^–5^ s^–1^). For pyruvic acid, this difference is more pronounced. A hydroxyl radical reaction constant of 1.2 × 10^8^ M^–1^ s^–1^^66^ yields a rate of 4.3 × 10^–9^ s^–1^, considerably lower than the rate from the kinetic model (*k*_sink_ × [oxidants] = 5 × 10^–4^ s^–1^). Indeed, pyruvic acid can be expected to be efficiently removed through photolysis.^67^

#### Production of Organic Acids from Ambient-Collected BrC Samples

In the firewood smoke samples, initial concentrations of acetic acid were 148 and 176 μM for the November and December samples, respectively (Table S5). During irradiation, there was an acetic acid production of 27 μM (November) and of 18 μM (December) (Figure 3), accounting for up to 2.2% of the initial TOC concentrations (Figure 2). Interestingly, formic acid was produced in higher quantities in the December sample, despite TOC concentrations being 50% lower compared to the November sample. Extended irradiation times up to 25 h also demonstrated further evolution of acetic and formic acid concentrations for the firewood experiments (Figure S12). Pyruvic acid and oxalic acid were produced at concentrations below 2 μM, representing a small fraction of the carbon mass balance (Table S5 and Figure 4). Compared to (NH_4_)_2_SO_4_-methylglyoxal, the overall production of organic acids appeared to occur at a slower rate in the firewood smoke samples. This observation could indicate that the organic matter is more photorecalcitrant, corroborating a similar hypothesis made based on the TOC analysis (Figure 2). Furthermore, the ambient firewood smoke aerosols were located at the source of the fire, better representing time point zero in a biomass burning plume.^68^ The interconnected effects of evolving chemistry and dispersion of these plumes further entangle the role of the photomineralization mechanism.

The initial concentration in the Padua sample was similar to (NH_4_)_2_SO_4_-methylglyoxal at 332 μM (Table S5). During photo-oxidation of the Padua sample, the production of organic acids was significantly lower compared to the other samples tested, consistent with the low extent of photomineralization for this sample observed in TOC analysis. Acetic acid was measured at high concentrations in the nonirradiated sample (268 μM) and increased by approximately 20 μM over 8 h of UVB exposure (Table S5 and Figure 3). Formic acid appeared to increase as well, though the magnitude of this change was small (2 μM). Both oxalic acid and pyruvic acid were below the limit of quantification (1 μM). The low rate of organic acid production observed here match the constant organic carbon concentrations reported in Figure 2 and confirm the organic matter in this sample to be highly photorecalcitrant. In addition, the high concentrations observed in the ambient aerosol samples could have originated from partitioning of ambient gaseous acetic acid to the liquid phase. Indeed, acetic acid has been observed to be ubiquitous in polluted urban environments^69^ as well as in biomass burning plumes,^70^ supporting the higher initial acetic acid concentrations observed.

#### CO and CO_2_ Production

CO and CO_2_ are the key oxidation products supporting a photomineralization mechanism and a decrease in TOC with UVB irradiation.^5,71^ These end products were quantified using a GC-FID setup, and samples analyzed were the second and third batch of (NH_4_)_2_SO_4_-methylglyoxal, the December sample for firewood smoke and the Padua Friday sample. SRFA results are reported in Borduas-Dedekind et al.^5^

**Figure 5:**
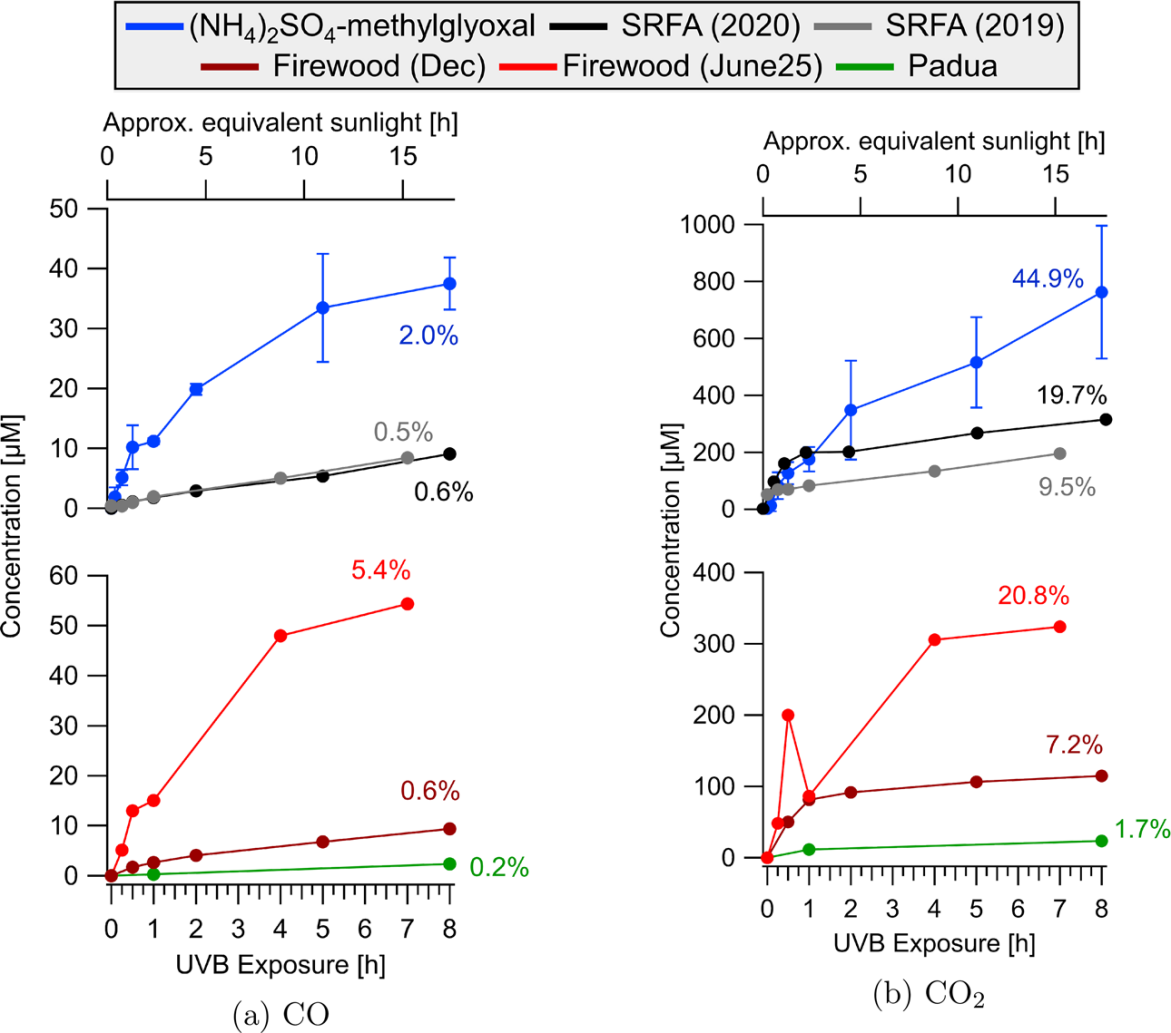
CO (a) and CO_2_ (b) concentrations measured by GC-FID as a function of UVB irradiation for the four types of BrC samples. Top panels and bottom panels show results for the lab-generated and field-collected BrC samples, respectively. The (NH_4_)_2_SO_4_-methylglyoxal solution time points were averaged and standard deviations are shown, as we expected this laboratory-generated sample to give reproducible results. Percentages indicate the maximum yield of the acid produced over 8 h from the initial TOC content in Figure 2. Additional experiments were conducted over 25 h of irradiation and show continued increases in concentration reaching up to 50% of TOC (Figure S13)

In (NH_4_)_2_SO_4_-methylglyoxal solutions, CO_2_ concentrations increased on average by 762 μM (Figure 5). On average, 37 μM CO was produced (Figure 5). The combined production of CO and CO_2_ exceeded the observed losses of organic carbon, as measured by TOC analysis (Table 1). Consequently, we interpret the TOC results to be underestimates and note the need for further detailed mechanistic studies to reconcile this mass balance discrepancy. The production of CO and CO_2_ observed via GC-FID analysis is likely a more accurate measure of total photomineralization for the BrC sample.

In the December firewood smoke sample, CO_2_ concentrations increased from 50 μM (Table S5) to 115 μM during UVB irradiation (Figure 5). The photomineralization to CO_2_ of the December firewood BrC appeared to be rapid at first, then slowed after 1 h (Figure 5). A similar trend was observed for CO, which increased by 10 μM. These results indicate an initial fast mineralization of a fraction of the organic matter, concurrent with the observed decrease in absorbance for this sample (Figure 1). Concentrations of CO and CO_2_ continued to increase over extended irradiation time, indicating sustained photodegradation (Figure S13). Thus, the production of CO and CO_2_ during the initial phase of UVB exposure are potentially linked to the degradation of photolabile chromophores.

For the Padua Friday sample, the observed production of CO and CO_2_ added up to 26 μM. While this change is small, these results indicate that photomineralization did in fact take place, albeit at a lower rate not captured by the TOC analysis.

### CCN Activity of BrC Samples

**Figure 6:**
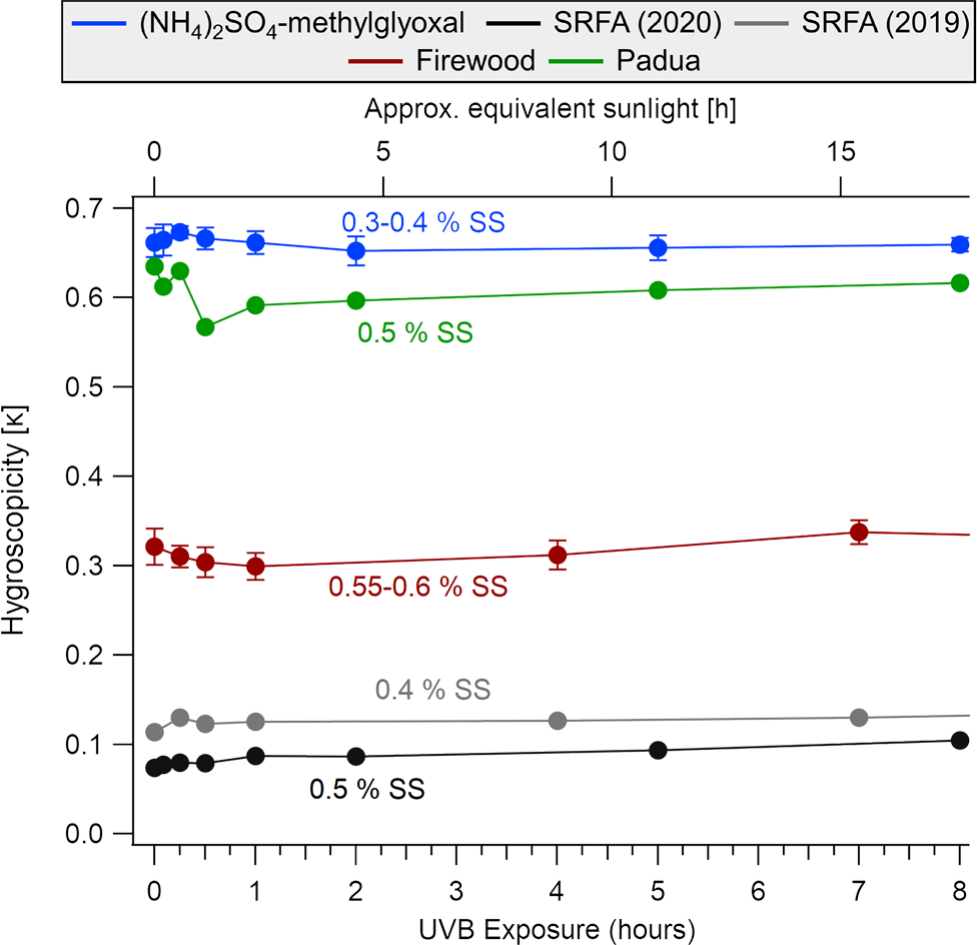
Changes in hygroscopicity represented by κ values as a function of UVB exposure and equivalent sunlight exposure. The BrC solution and firewood samples depicted are averages of three experiments each with standard deviations (more specifically, the firewood samples are from the June, July, and August samples). The CCNC was operated at 0.30−0.40% SS, 0.40 and 0.50% SS, 0.55−0.60% SS and 0.5% SS for the (NH_4_)_2_SO_4_-methylglyoxal, SRFA, firewood, and Padua samples, respectively.

The range of BrC samples, namely, (NH_4_)_2_SO_4_-methylglyoxal, SRFA, firewood smoke, and ambient Padua aerosol solutions, led to a range of CCN abilities driven by the salt content of the sample. The hygroscopicity of the BrC samples is reported using the hygroscopicity parameter κ and reported over a period of 8 h of irradiation, corresponding to 1.5 days (17.6 h) in the atmosphere (Figure 6). These results were not dependent on irradiation time since additional experiments conducted over 25 h of irradiation led to similar results (Figure S18).^5^

(NH_4_)_2_SO_4_-methylglyoxal was the most hygroscopic material with κ values between 0.58 and 0.65 for the nonirradiated samples. This κ value is similar to that of pure (NH_4_)_2_SO_4_ particles, measured during calibration of the instrument (κ = 0.59–0.67) (Figure S17b). No significant change in κ was observed in response to UVB irradiation (Figure 6), indicating that the chemical changes in (NH_4_)_2_SO_4_-methylglyoxal due to photomineralization did not translate to a change in CCN activity. In fact, the CCN ability is solely driven by the salt content and thus changes in κ due to photomineralization of the organic mass is insignificant. Nonetheless, surface-active organics, such as methylglyoxal and acetaldehyde, can reduce the surface tension of aerosols upon uptake, and this effect is largest for aerosols containing salts.^56^ To elucidate the effect of this surface tension depression on cloud activation, Sareen et al.^72^ exposed (NH_4_)_2_SO_4_ aerosols suspended in a smog chamber to gaseous methylglyoxal. The uptake of methylglyoxal was observed to increase the κ values of the (NH_4_)_2_SO_4_ aerosols from 0.60 up to 0.81 over 5 h of methylglyoxal exposure. This increase in hygroscopicity seems to arise from a complex interplay of simultaneous changes to the water activity (Raoult) term and the curvature (Kelvin) term exerted by the presence of organic surfactants.^73^ However, Sareen et al.^72^ further reported that atomized bulk solutions of (NH_4_)_2_SO_4_-methylglyoxal did not exhibit the same effect as observed in the smog chamber experiments. As a consequence, the photomineralization of the organic components in bulk mixtures of (NH_4_)_2_SO_4_-methylglyoxal may have a negligible effect on the CCN ability of the atomized aerosols.

Next, the κ values of the aerosolized solutions of SRFA ranged between 0.07 and 0.10 at 0.5% SS (Figure 6). This BrC sample had the lowest κ values measured of the four types of samples, consistent with this material having the highest organic carbon to salt ratio. An increase of κ values from 0.11 to 0.13 was observed as a function of photomineralization, consistent with other DOM samples measured by Borduas-Dedekind et al.^5^

The firewood smoke aerosols were moderately hygroscopic with a κ value of 0.36 and 0.28 for the November and December samples, respectively. Aerosols from biomass burning emissions vary widely in hygroscopicity owing to the complex nature of their chemical composition, source and time from emission. Engelhart et al.^74^ reported κ values ranging from 0.06 to 0.6 for organic aerosols formed from the combustion of 12 different types of biomass fuels, a similar range observed by Petters et al.^75^ The κ values of birch wood smoke aerosols measured correspond to this range, but are slightly higher than those of pine and spruce fuels (κ = 0.06–0.21) reported in the aforementioned studies. In addition, Mukherjee et al.^23^ observed κ values below 0.17 for BrC samples from combustion of charcoal, wood, leaf, cow dung, and grass. For our firewood smoke samples, the change in CCN ability in response to photochemical aging increased compared to (NH_4_)_2_SO_4_-methylglyoxal solutions. Unlike (NH_4_)_2_SO_4_-methylglyoxal solutions, chemical changes such as photobleaching continued to occur throughout the full length of UVB irradiation. The organic matter in firewood smoke, more specifically the chromophores, photodegraded at slower rates than the (NH_4_)_2_SO_4_-methylglyoxal solutions. The photomineralization mechanism did not significantly alter the CCN ability of the firewood smoke organic aerosols.

Lastly, the sample collected in Padua was highly hygroscopic with a κ value of 0.64 for the nonirradiated sample. The Padua sample had a low conductivity (≈21 μS cm^–1^) yet had significant concentrations of NH_4_^+^ and NO_3_ (Table S3). Like (NH_4_)_2_SO_4_-methylglyoxal, no significant changes were observed in response to UVB irradiation, as expected based on the low extent of photomineralization observed for this sample. Previously, Rosati et al.^76^ reported an average κ value of 0.22 for the aerosols in the Po Valley mixed layer, significantly lower than in our experiments. It is worth noting that this value corresponds to a field campaign in summertime. Additionally, Psichoudaki et al.^77^ reported κ values between 0.15 and 0.25 for urban ambient aerosols in wintertime in Athens, Greece, likely to be heavily influenced by biomass burning emissions. Furthermore, Burkart et al.^78^ found no significant seasonal variation in κ for urban aerosols in Vienna, indicating that seasonal differences are unlikely to account for the difference in the Padua sample κ to the Po Valley-average κ value of 0.22 reported by Rosati et al.^76^ To explain the hygroscopic properties of the Padua samples, a more detailed analysis of the chemical composition would be required. Nonetheless, we identified the photomineralization mechanism across all BrC samples and estimate the atmospheric relevance of lab-generated BrC.

## Atmospheric Implication of the Photomineralization Mechanism in BrC Samples

The photochemical changes quantified across four different BrC samples provide evidence for the photomineralization mechanism. The photomineralization rates were fastest for the lab-generated BrC samples and slowest for the ambient BrC samples (Table 1). The changes observed include an overall loss of organic carbon, albeit not observed in the ambient Padua sample, a decrease in absorbance, a decrease in pH, an increase in conductivity, the production of organic acids and concurrent production of CO and CO_2_. In addition, all samples exhibited photobleaching although at different rates, indicating the degradation of chromophores. The (NH_4_)_2_SO_4_-methylglyoxal solution had the highest absorbance at 20 mg C L^–1^ and photobleached with the fastest rates (Figure 1 and Table 1). The loss of organic mass and production of organic acids were most pronounced in the (NH_4_)_2_SO_4_-methylglyoxal solution and occurred to a lesser extent in the firewood smoke samples. In the sample obtained in Padua, photobleaching as well as the production of CO, CO_2_, and acetic acid were observed at low rates, consistent with lower absorbance and fewer chromophores. Overall, the carbon mass balance is not fully accounted for; the observed production of CO_2_ was up to 50% of the initial TOC, but the measured TOC only decreased by around 10%. We know that the TOC analysis underestimates organic carbon (see the SI for discussion on lignin) as well as the possibility of forming insoluble inorganic carbon; thus, further research is warranted to describe the mechanism of photoproduct generation.

Despite the photochemical changes, CCN abilities did not change substantially for the range of BrC samples. The CCN abilities were dictated by the salt content of the BrC solution, arguably masking the photomineralization effect on the CCN abilities. Solutions of (NH_4_)_2_SO_4_-methylglyoxal, SRFA, firewood smoke, and ambient Padua samples had hygroscopicity parameters κ of 0.6, 0.1, 0.3, and 0.6, respectively. As expected, the SRFA solution had κ 0.1 and was most impacted by the photomineralization mechanism, and thus, the photomineralization mechanism will be most important for organic aerosols with little salt content.

The choice of BrC sample for research in atmospheric chemistry is judicious and should be placed in context when extrapolating to ambient photochemical aging. The types of molecules and the matrix effects in firewood smoke and urban PM are more complex and varied than in (NH_4_)_2_SO_4_-methylglyoxal and SRFA. Consequently, a larger number of chemical transformations are necessary to yield CO and CO_2_, resulting in an overall lower rate of photomineralization over longer time scales (Table 1). This conclusion can also be inferred from the observed production of organic acids across the different samples. The degradation of chromophores and associated photobleaching was most substantial in (NH_4_)_2_SO_4_-methylglyoxal, but occurred at a slower rate in firewood smoke BrC solutions. Of all BrC samples tested, the ambient urban PM from Padua showed the least evidence of photomineralization, with little change in organic carbon concentration and low production of CO, CO_2_ and organic acids (Table 1). Of note, our experiments with four different types of BrC solutions were carried out in bulk solutions, and not in aerosol droplets. There is growing evidence that droplet chemistry can have different kinetics due to differences in gradients in pH, ionic strength and morphology.^79−81^ Ultimately, the range of chemical composition of the four types of BrC samples should help us reflect on our choices of BrC proxies and samples in our laboratory experiments.^19^

## Data Availability

The data in this article is available open access through the Zenodo repository at DOI: 10.5281/zenodo.7057970.

## Supporting Information Available

Methods, TOC measurement issues, data for lignin, photochemistry experiments, aerosol–cloud interaction experiments (PDF)
Additional data for (NH_4_)_2_SO_4_-methylglyoxal, firewood smoke, Padua, and SRFA samples (XLSX)
