# Supplementary material for: Tracking the Photomineralization Mechanism in Irradiated Lab-Generated and Field-Collected Brown Carbon Samples and Its Effect on Cloud Condensation Nuclei Abilities
Source: ACS Environ Au. 2023 Mar 17;3(3):164–78. doi: 10.1021/acsenvironau.2c00055 (PMC10197166; doi:10.1021/acsenvironau.2c00055)
Supplement: Supplementary file 1 — vg2c00055_si_001.pdf [file vg2c00055_si_001.pdf]

Supplemental Information: Tracking the photomineralization mechanism in irradiated lab-generated and field-collected brown carbon samples and its effect on cloud condensation nuclei abilities

Silvan Müller,[†,‖] Chiara Giorio,[‡,¶] and Nadine Borduas-Dedekind[*,†,§]

†*Department of Environmental Systems Science , ETH Zurich, Zurich, 8092, Switzerland*

‡*Yusuf Hamied Department of Chemistry, University of Cambridge, Cambridge, CB2 1EW, United Kingdom*

¶*Department of Chemical Sciences, University of Padova, Padova, 35131, Italy*

§*Department of Chemistry, University of British Columbia, Vancouver, V6T 1Z1, Canada*

‖*Now at: Ecosens AG, Wallisellen, 8304, Switzerland*

E-mail: borduas@chem.ubc.ca

Phone: +1 604-822-4435

Methods

Brown carbon sample preparation, collection and characterization

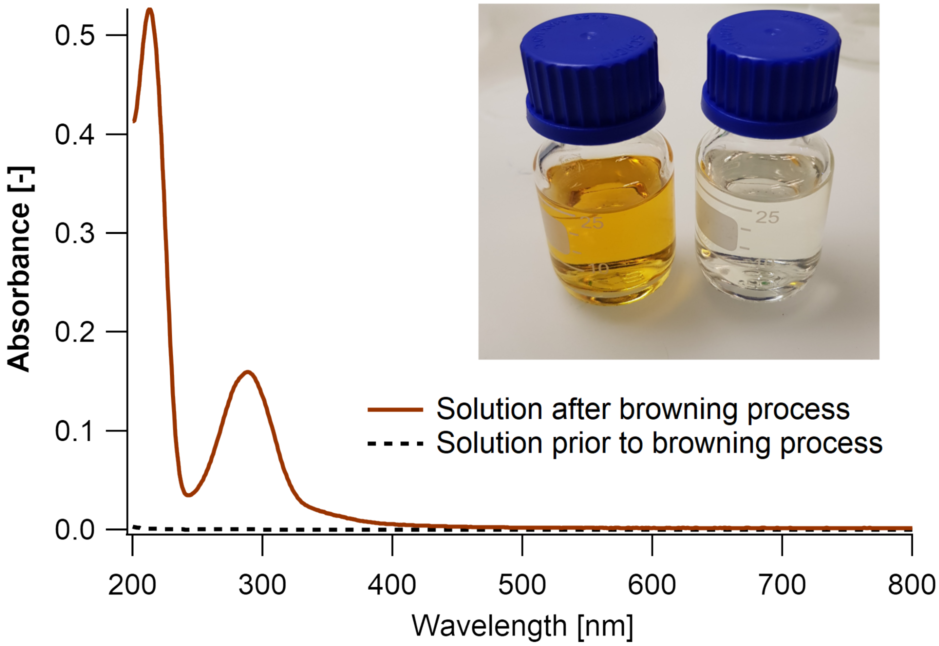

Figure S1: Absorbance of ammonium-sulfate methylglyoxal solutions before (colorless) and after (yellow-brown) the two-week browning process in the dark.

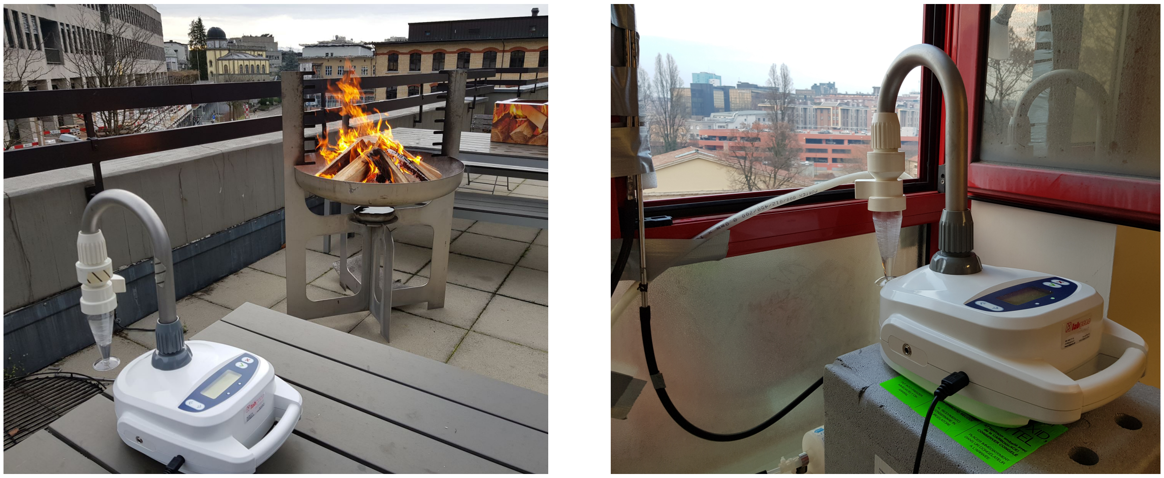

(a) (b)

Figure S2: Photographs of the aerosol collection experimental setups using the *Coriolis* μ sampler. (a) Birch wood logs were burned in a round brazier bowl on 11 November (pictured) under dry, cloudy conditions and collected as firewood smoke. The *Coriolis* μ was placed downwind two meters away from the fire. (b) Urban wintertime particulate matter was sampled at the Department of Chemical Sciences of the University of Padua, in Padua, Italy, close to the city center. The *Coriolis* μ was placed at an open window to collect undiluted outdoor air.

Table S1: Overview of the firewood experiment dates, details and sampling conditions. During the collection time, the Coriolis cone was refilled to the 15 mL mark with Sigma-Aldrich or Milli-Q water. Each 20 mins, the Coriolis cone was switched with a pre-cleaned cone (see Methods sections in the main text). The TOC concentrations reflects the combined TOC from all the sampling cones of firewood smoke for the experiment day. GC-FID experiments were operated under sealed gas conditions and were therefore exclusively used for these measurements. Due to periodic problems with the TOC analyzer, TOC data before photochemical experiments are available for all samples, but photochemical timepoints are not available for the June11 and Aug sample (noted with a star).

**Sample Name**	**Collection Date**	**Collection Time**	**TOC**	**TOC (diluted)**	**Experiments**
	d-m-y	mins	mg C/L	mg C/L	
**November**	11.11.2019	80	130.6	47.3	Photochemistry, CCNC, IN
**December**	11.12.2019	120	45.2	21.5	Photochemistry, CCNC, IN
**May**	28.05.2020	140	81.7	20.4	GC-FID
**June11**	11.06.2020	240	84.7*	22.5*	GC-FID, Photochemistry, CCNC, IN
**June25**	25.06.2020	240	46.8	20.0	GC-FID
**July**	09.07.2020	240	133.9	20.0	Photochemistry, CCNC, IN
**Aug**	06.08.2020	240	102.7*	20.1*	Photochemistry, CCNC, IN

Table S2: Overview of ambient air aqueous samples collected in Padua, Italy from January 13 to 17, 2020. Times represent collection intervals, which included Milli-Q water refills to the 15 mL mark on the Coriolis cone every 10 mins. The sample name represents 2 or 3 combined samples. In a subsequent filtration step, the solutions were diluted to 50 or 100 mL using nanopure water and analyzed for their total organic carbon (TOC) content. Only the Friday samples were combined into a minimum volume for a maximum TOC content. Therefore, the Friday sample was used as a BrC sample in this work since it was the only sample with enough material required for all the photomineralization experiments.

**Date**	**Time**	**Sample Name**	**# of Samples**	**Post-Filtration Volume**	**TOC** [$mg\,C\,L^{-1}$]
Monday 13.01.2020	09:50-10:30 13:15-17:45	*Monday*	3	100 mL	4.1
Tuesday 14.01.2020	08:30-12:50	*Tuesday AM*	2	50 mL	4.4
	13:00-18:45 19:45-22:00	*Tuesday PM*	3	100 mL	5.0
Wednesday 15.01.2020	08:30-12:45	*Wednesday AM*	2	50 mL	6.7
	12:45-19:00	*Wednesday PM*	3	100 mL	4.9
Thursday 16.01.2020	08:15-12:45	*Thursday AM*	2	50 mL	5.1
	12:45-19:15	*Thursday PM*	3	100 mL	4.8
Friday 17.01.2020	08:00-12:15 15:00-17:15	*Friday*	3	50 mL	67.4

Table S3: Ion chromatography data of the PM sample collected in Padua, Italy

**Ion**	**Concentration (ng/m^3)**
Na^+	251
NH_4^+	3394
K^+	2102
Mg^{2+}	52
Ca^{2+}	407
Acetate	69
Formate	133
MSA	120
Cl^-	556
NO_2^-	8
NO_3^-	20509
Malonate	563
Sulphate	3220
Oxalate	206
Phosphate	44

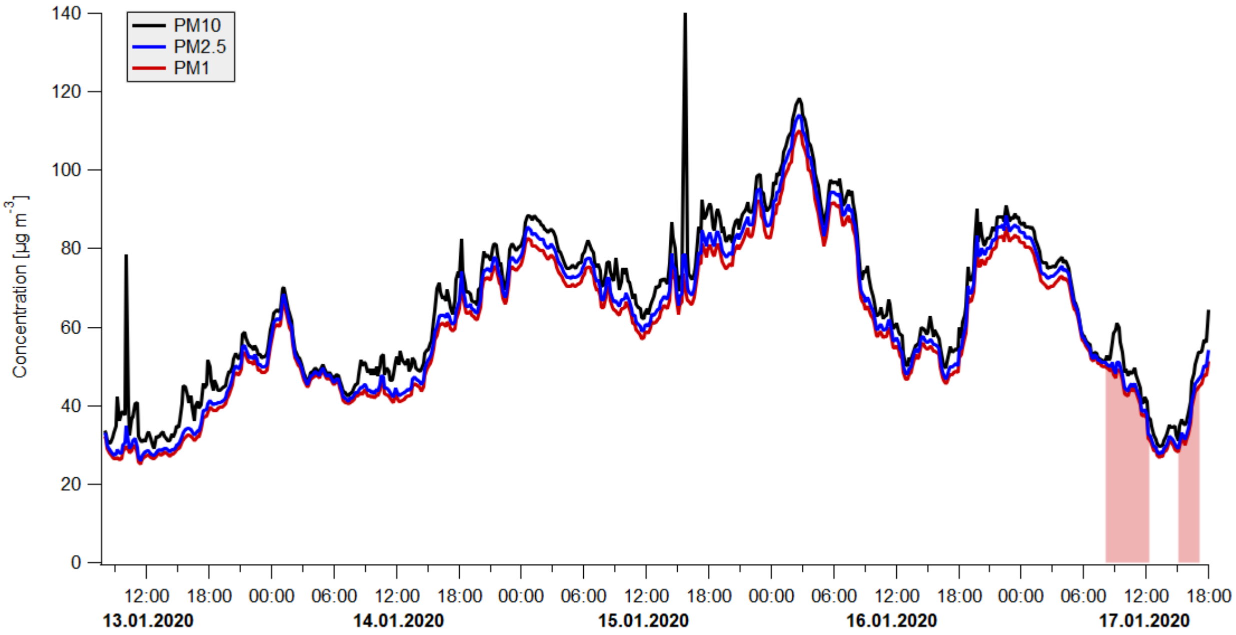

Figure S3: Concentrations of ambient PM_1, $PM_{2.5}$ and PM_{10} measured by an optical particle counter during the sampling period with the Coriolis in Padua from 13.01 to 17.01.2020. Note that concentrations peaked during nighttime. The shaded areas indicate the collection times of the *Padua Friday* sample which was used for photochemical experiments in this work. This sample had the highest concentration of organic carbon of all samples collected because of combined samples into a minimum volume, even though the concentration of particulate matter on that day was at the lower end of the range observed throughout the week.

TOC measurement issues

An alternative explanation for the low loss of organic carbon is an insufficient recovery in the TOC analyzer for the non-irradiated firewood smoke sample compared to the irradiated samples. Indeed, we have accumulated evidence that organic carbon in complex molecules is undercounted by the Shimadzu TOC analyzer employed. For example, UVB irradiation of lignin led to an observed increase in TOC over time (Figure S4). This unexpected result could have been caused by photomineralization of organic carbon being offset by an increasing recovery rate due to the concurrent degradation and fragmentation of lignin. As firewood smoke likely contains a substantial fraction of complex organic molecules like lignin (further evidenced by similar MAC spectra), this TOC recovery effect could have masked some of the loss of organic carbon. This hypothesis is supported by the production of CO and CO_2 observed for the *December* sample (134 µM or $1.6\,\mathrm{mg\,C\,L^{-1}}$), which far exceeded the concurrent loss of organic carbon. Conversely, the mass balance was consistent for SRFA and ammonium sulfate-methylglyoxal. Accounting for the production of CO and CO_2 as a measure of mineralization for the *December* firewood smoke sample, instead of results from TOC analysis, yields an organic carbon loss of $1.6\,\mathrm{mg\,C\,L^{-1}}$ which elevates the overall organic carbon conversion from 2.4% to 8.4%. Thus, it can be assumed that the mineralization rates reported in Figure S7 represent a lower end estimate.

Lignin

Furthermore, preliminary experiments were conducted with lignin (Sigma-Aldrich), a complex polymer present in all terrestrial plants. Using nanopure water, $20\,\mathrm{mg\,C\,L^{-1}}$ solutions were prepared and exposed to UVB irradiation for a duration of 4 hours and for 25 hours, respectively, in two Rayonet experiments. Further analyses included TOC measurements and UV-Visible spectroscopy. While these experiments were not intended to be included in the main part of this thesis, the results provided useful insights into the absorbance fea-

tures of the firewood smoke samples, and helped identify a potential problem with the TOC analyzer.

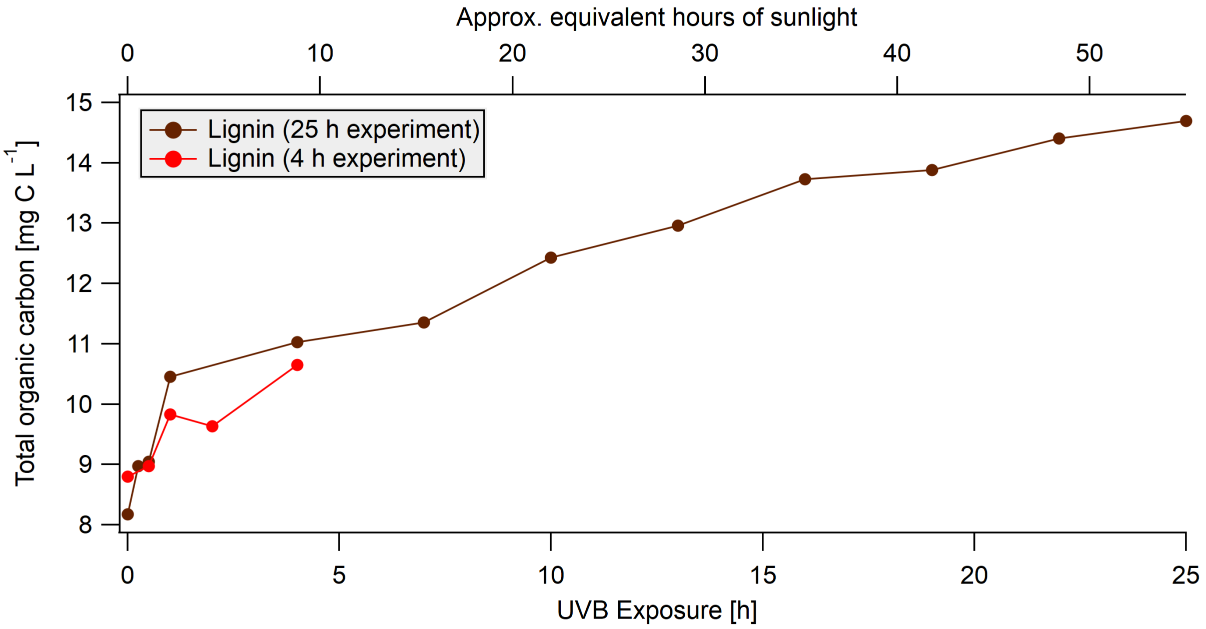

Figure S4: In two separate experiments, total organic carbon (TOC) concentrations were quantified in lignin solutions exposed to UVB radiation for 4 hours (red) and for 25 hours (brown). In both experiments, the solutions were prepared such that they contain 20 mg C L^{-1} of organic carbon, but the measurement under-reported these concentrations by over 50%. Furthermore, the TOC measurement showed an increasing trend over time. These results were interpreted as evidence for the insufficient recovery rate of complex molecules like lignin in the TOC analyzer. Conversely, increasing TOC concentrations over time stem from a higher degree of fragmentation and degradation in these molecules due to UVB irradiation.

Photochemistry experiments

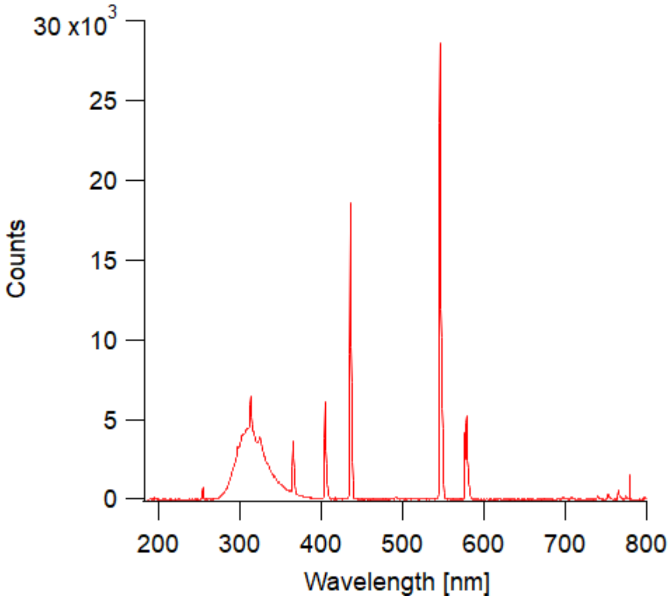

Figure S5: A portable spectrophotometer (Ocean Optics) was used to determine the wavelength-dependent photon flux between 200 and 800 nm in the photoreactor with 6 UVB bulbs peaking at 300 nm.

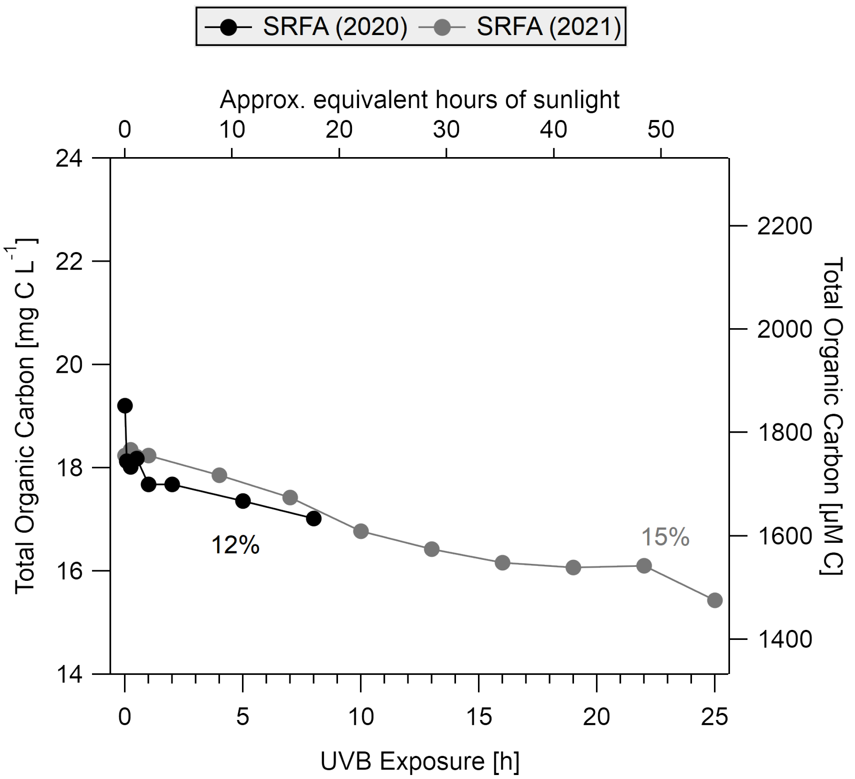

Figure S6: TOC loss as a function of UVB irradiation for SRFA samples.

Table S4: Values of the parameters used in the kinetic box model for formic acid and pyruvic acid. The relevant equation is displayed at the bottom of the table. $[formic/pyruvic\ acid]_0$ is the concentration of formic acid or pyruvic acid in the non-irradiated sample. γ and k_{OC} are the yield and rate constant, respectively, for the production of these acids from the photooxidation of organic compounds; here a value of $5.6 \times 10^9\,\mathrm{M^{-1}s^{-1}}$ was used for k_{OC} based on the reaction rate constant of hydroxyl radicals with organic molecules[2]. $[oxidants]$ is the concentration of oxidants, and $[TOC]$ the concentration of organic matter available for formic acid/pyruvic acid production, assumed to be equal to the approximate organic carbon concentration of the solutions in UVB irradiation experiments (i.e. $20\,\mathrm{mg\,C\,L^{-1}}$). k_{sink} combined with $[oxidants]$ represents the idealized sum of all sink processes by which formic acid was depleted. γ, $[oxidants]$ and k_{sink} were optimized until the fit represented well the experimental data.

	**Formic acid**	**Pyruvic acid**	Basis of values
$[formic/pyruvic\ acid]_0$	$3.95 \times 10^{-5}\,\mathrm{M}$	$2.12 \times 10^{-6}\,\mathrm{M}$	Ion chromatography measurement
$[TOC]$	$1.6 \times 10^{-3}\,\mathrm{M}$	$1.6 \times 10^{-3}\,\mathrm{M}$	$\approx 20\,\mathrm{mg\,C\,L^{-1}}$
γ	0.14	0.04	Optimized to experimental data
k_{OC}	$5.6 \times 10^9\,\mathrm{M^{-1}s^{-1}}$	$5.6 \times 10^9\,\mathrm{M^{-1}s^{-1}}$	[2]
k_{sink}	$4 \times 10^8\,\mathrm{M^{-1}s^{-1}}$	$1 \times 10^{10}\,\mathrm{M^{-1}s^{-1}}$	Optimized to experimental data
$[oxidants]$	$5 \times 10^{-14}\,\mathrm{M}$	$5 \times 10^{-14}\,\mathrm{M}$	Optimized to experimental data

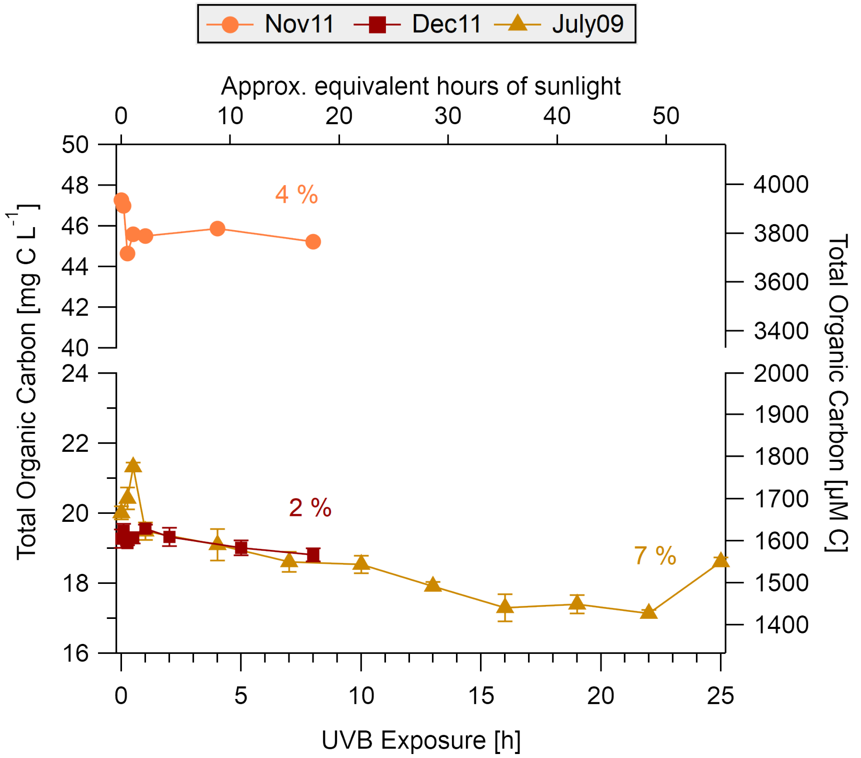

Figure S7: TOC loss as a function of UVB irradiation for three firewood smoke solutions for comparison with longer photochemical exposure times. Loss of TOC is relatively slow, with some fluctuations, in the firewood smoke samples compared to SRFA loss. See Table S1 for sampling details of each firewood sampling experiment.

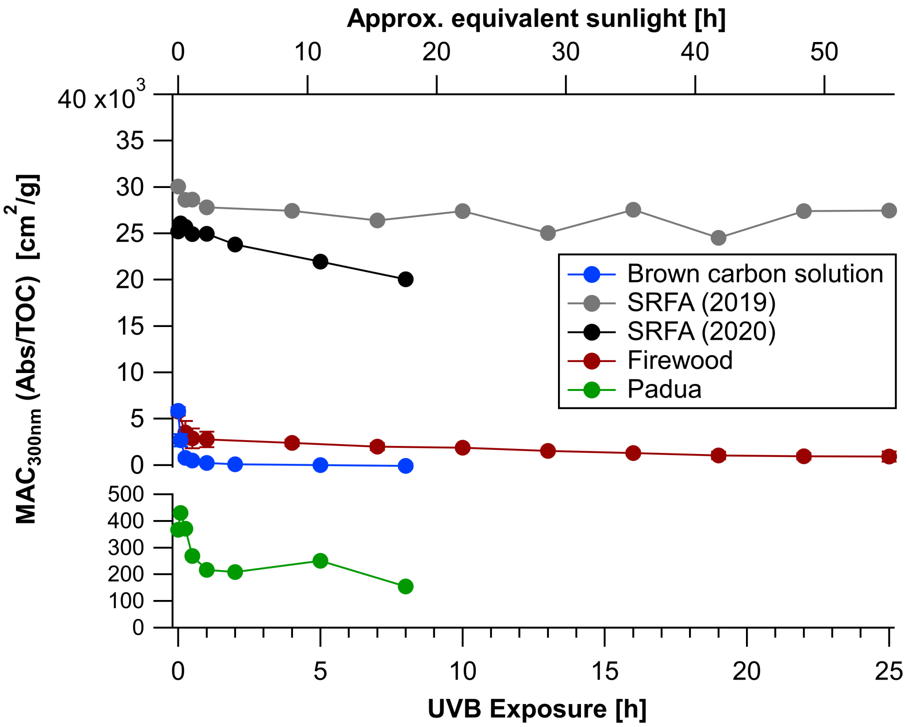

Figure S8: Changes in MAC_{300nm} as a function of UVB irradiation for extended lengths of time for all types of BrC solutions. Brown carbon solution is the ammonium sulfate methyl glyoxal solution and the SRFA (2019) sample is from Borduas-Dedekind et al.[1]

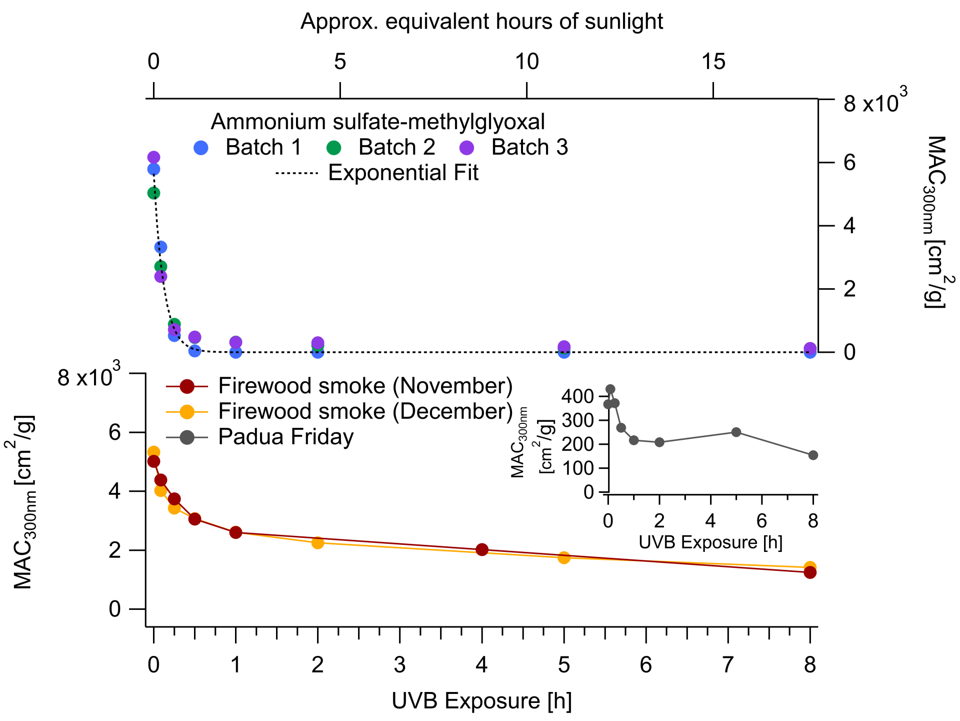

Figure S9: Changes in MAC_{300nm} during UVB irradiation experiments. Data for *Padua Friday* is shown as an inset due to the difference in magnitude to the firewood smoke samples. The absorbance of ammonium sulfate-methylglyoxal and firewood smoke appeared to decrease exponentially. To obtain the rate of photobleaching, exponential fits were added, yielding a first-order rate constant of $4.02\,h^{-1}_{sunlight}$ for ammonium sulfate-methylglyoxal and $82\,h^{-1}_{sunlight}$ for the firewood smoke *December* sample with respect to photobleaching at 300 nm.

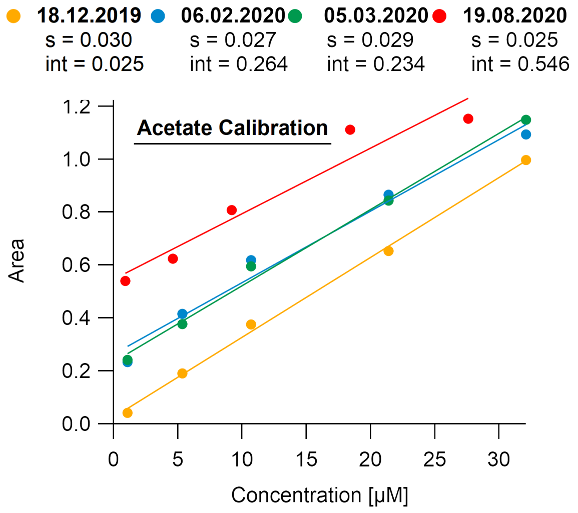

(a)

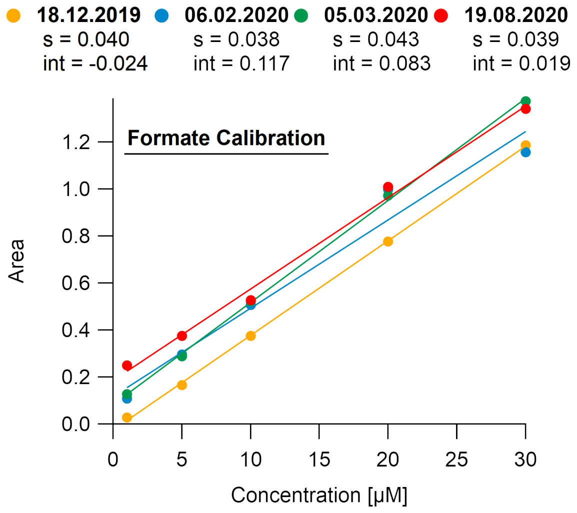

(b)

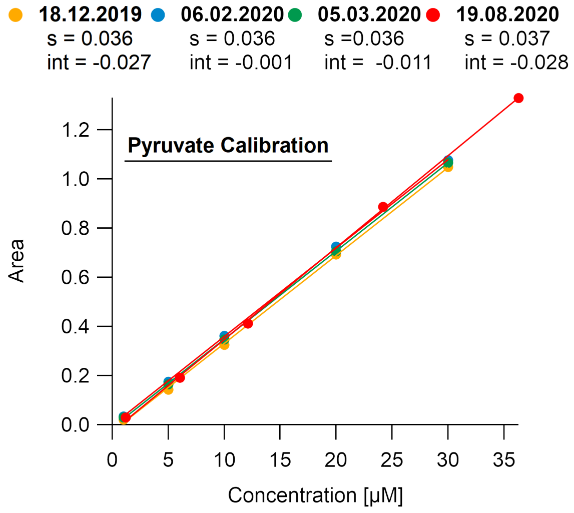

(c)

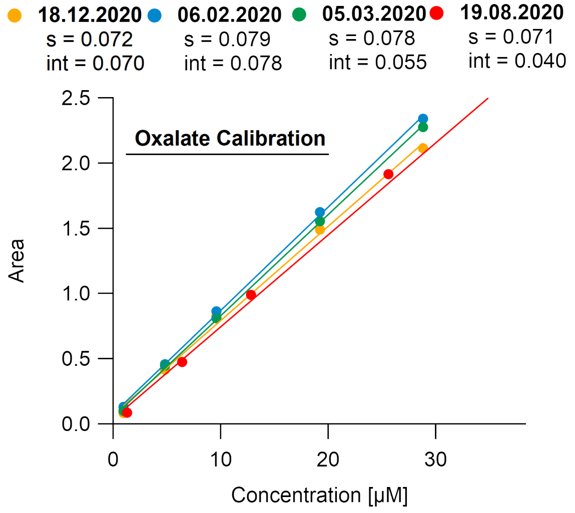

(d)

Figure S10: Ion chromatography (IC) calibrations of (a) acetic acid, (b) formic acid, (c) pyruvic acid, and (d) oxalic acid for 4 sets of IC calibrations over the course of this work. For each calibration, the slope (s) and intercept (int) are reported. Overall, the slopes were similar between calibrations and the most recent calibration was used to quantify the organic acids. On the other hand, the y-intercept of the acetate and formate calibrations drifted between measurements, most noticeably for acetate (panel (a)).

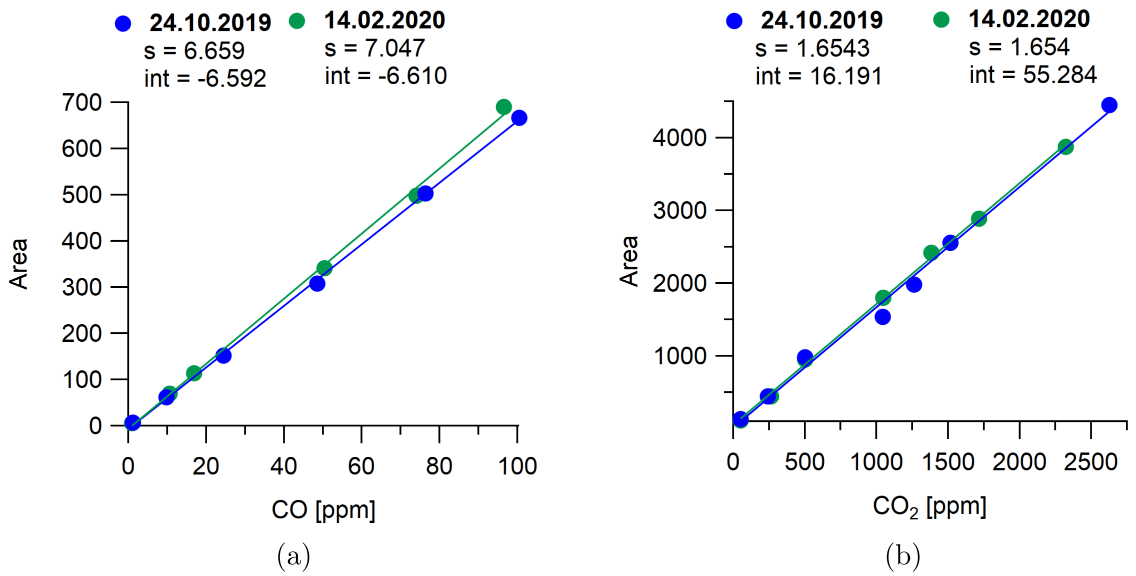

Figure S11: Calibration curves of CO and CO_2 on the GC-FID instrument. New gas standards from Messer (Germany) were prepared before each set of measurements (24.10.2019 and 14.02.2020). CO was calibrated between 0 and 100 ppm whereas CO_2 was calibrated between 50 and 2500 ppm. Dilutions of the standards were made using N_2 flushed vials; the exact concentrations were then calculated based on vial pressure measurements pre- and post-injection of the gas sample on the GC-FID.

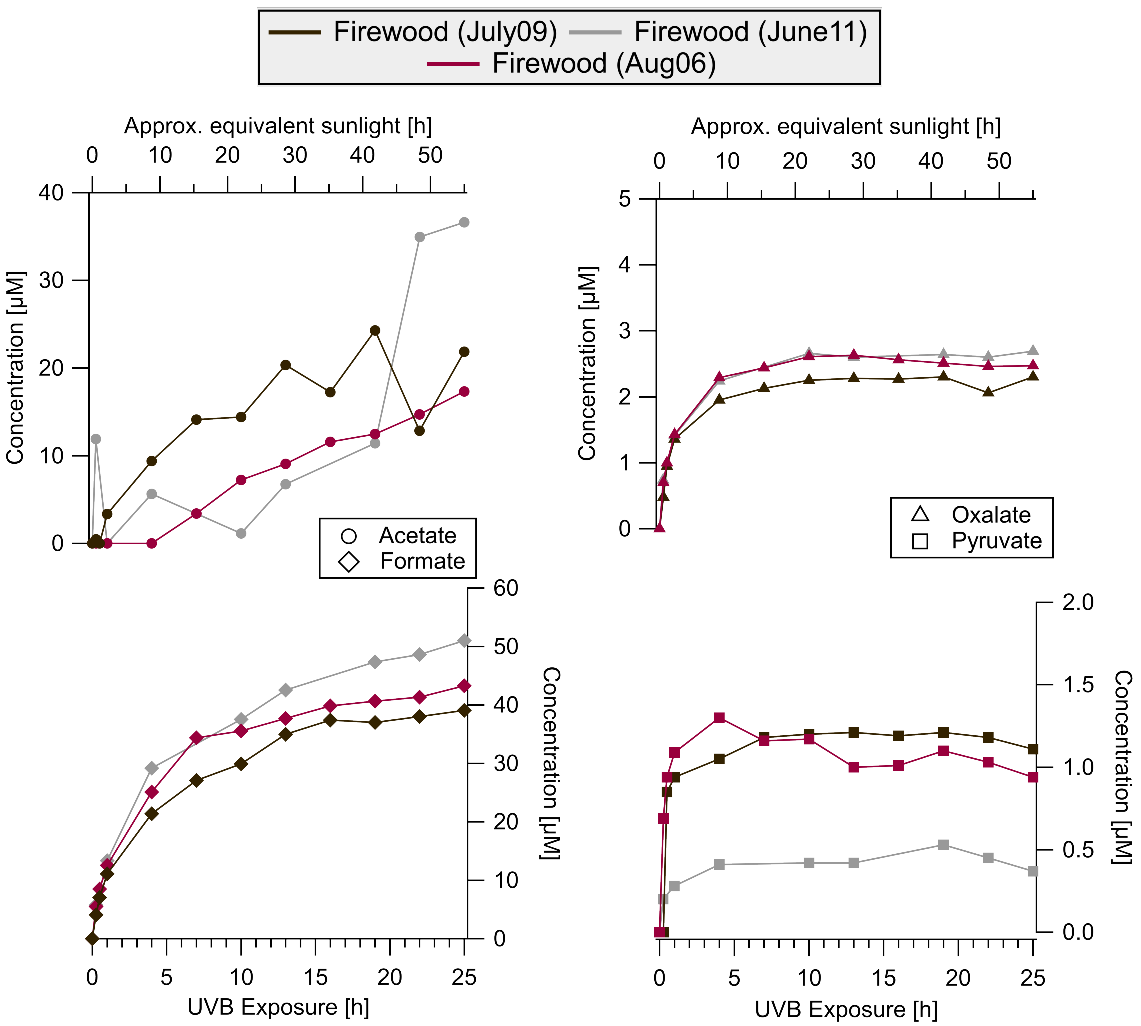

(a) Acetic and formic acids

(b) Oxalic and pyruvic acids

Figure S12: Acetic and formic acid (a), pyruvic and oxalic acid (b) concentrations as a function of UVB irradiation for the firewood BrC samples. The concentrations of the photoproducts continue to increase after prolonged irradiation times. This figure represents an extension of Figures 3 and 4 in the main text.

Table S5: The initial concentrations of CO, CO_2, and organic acids i the different BrC samples, measured by GC-FID and IC.

	CO µM	CO_2 µM	Acetic acid µM	Formic acid µM	Pyruvic acid µM	Oxalic acid µM
**$(NH_4)_2SO_4$-methylglyoxal**						
Batch 1	0.18	202.1	367.9	45.37	3.13	0.00
Batch 2	0.18	162.7	350.4	47.0	3.01	0.00
Batch 3	0.00	292.4	347.8	34.5	3.40	0.00
**Firewood samples**						
Nov	-	-	147.7	47.8	1.47	1.00
Dec	0.13	50.2	176.5	40.3	0.00	0.00
May28	0.58	130.0	-	-	-	-
June11	0.00	105.5	250.5	59.1	0.96	0.46
June25	0.00	88.6	-	-	-	-
July09	0.27	61.3	-	-	-	-
Aug06	-	-	219.2	51.5	0.00	0.00
**Padua**	0.11	125.9	332.1	4.6	0.00	0.00
**SRFA**	0.00	163.1	0.96	0.35	0.35	1.01

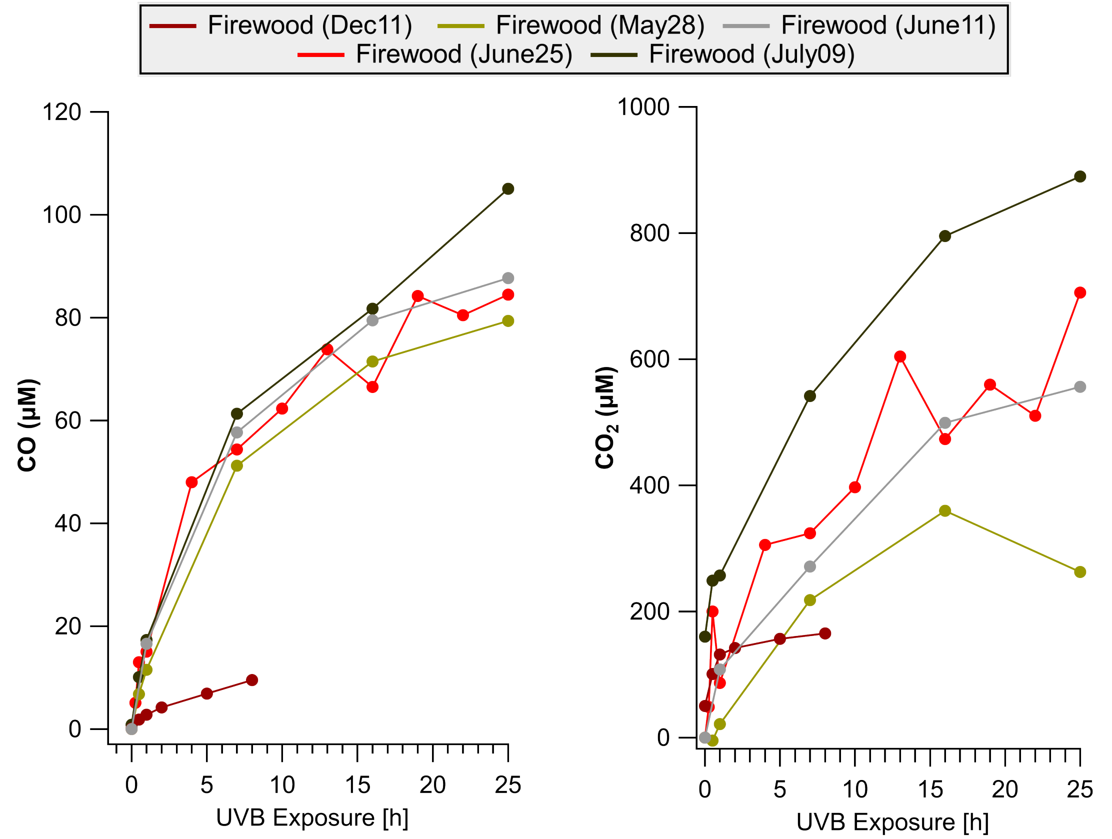

(a) CO

(b) CO_2

Figure S13: CO (a) and CO_2 (b) concentrations measured by GC-FID as a function of UVB irradiation for the firewood BrC samples. The CO and CO_2 concentrations continued to increase even after longer irradiation times. This figure represents an extension of Figure 5 in the main text.

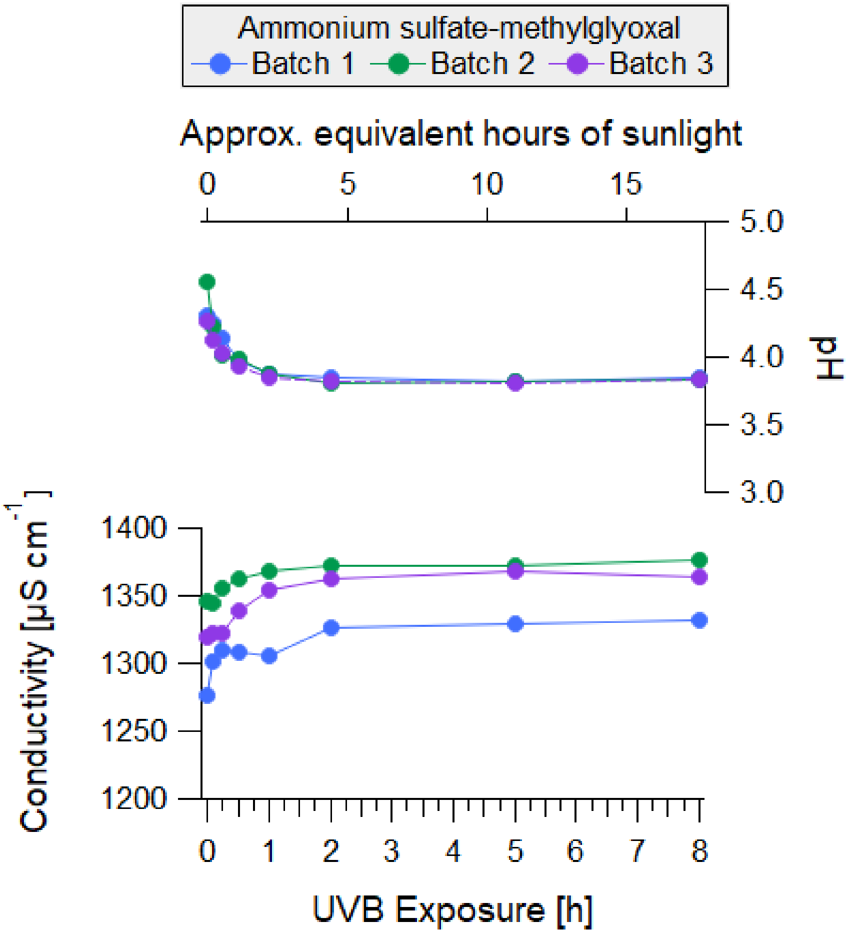

Figure S14: pH and conductivity values as a function of UVB irradiation for the ammonium sulfate methyl glyoxal samples

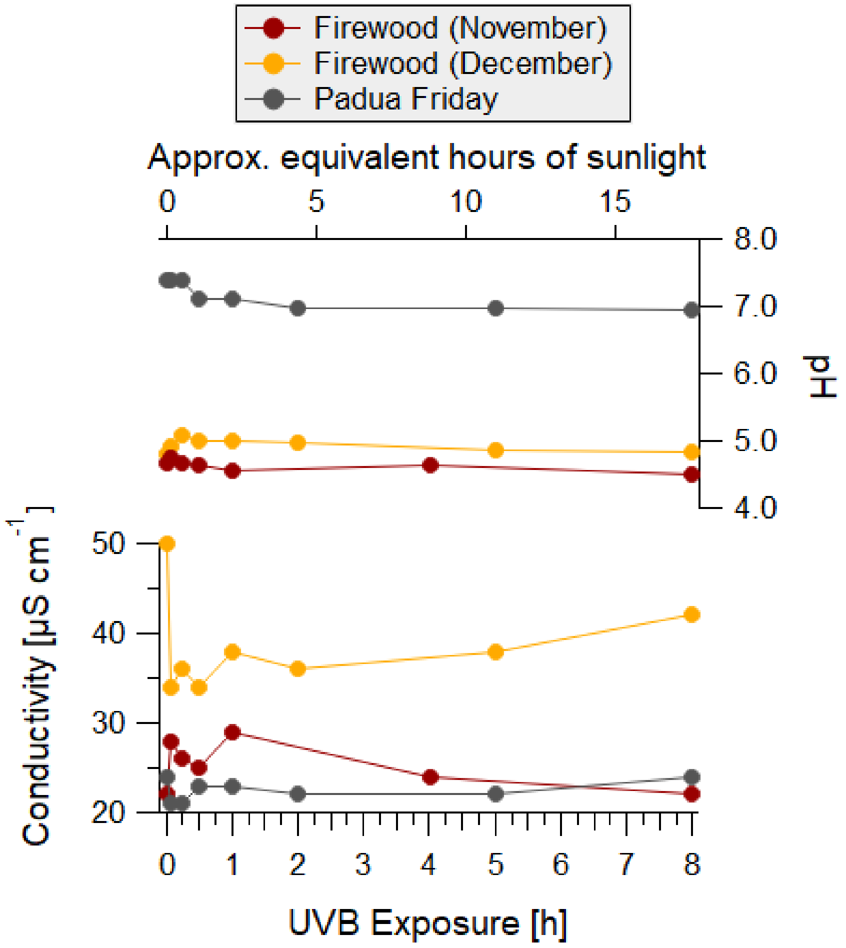

Figure S15: pH and conductivity values as a function of UVB irradiation for the Padua ambient samples

Aerosol-cloud interaction experiments

Cloud condensation nuclei

The flow of the CCNC was calibrated using a volumetric flow meter (Gilibrator-2, Sensidyne, USA). The effective water vapor pressure of the instrument was calibrated according to the procedure described by Rose et al.[3] which takes advantage of the well-characterized hygroscopic properties of ammonium sulfate particles. Aerosols generated from an ammonium sulfate solution (4.9 mM) were measured with the CCNC at six supersaturation conditions between 0.1% and 0.6% (Figure S17). For each supersaturation, the temperature in the CCNC growth column was monitored by built-in temperature sensors. The critical diameter for CCN activation, D_d, was obtained from the corresponding activation curve. With the critical diameter for ammonium sulfate particle activation and column temperature, a corresponding supersaturation can be calculated using the Aerosol Diameter Dependent Equilibrium Model (ADDEM) developed by Topping et al.[4]. This theoretical supersaturation was then compared to the value in the CCNC. Any offset between the two values was corrected such that the programmed supersaturation in the CCNC matches the one calculated via ADDEM (Figures S16 and S17). Unlike Rose et al.[3], no correction was made concerning doubly charged particles. Doubly charged particles can appear as a plateau on the left side of the activation curve (e.g. at the 0.1% supersaturation activation curve in Figure S17a). However, since we collected a large number of measurement points along the activation curve, there was no effect of the doubly charged particles on the quality of the sigmoidal fit (Figure S16a).

Additional control experiments included regulating the flow of air through the atomizer to ensure particle counts exceeded $1000\,cm^{-3}$. In between measurements, nanopure water was added to the atomizer to clear the system of residual material. This step was repeated, each time using fresh nanopure water, until the recorded particle counts at 40 nm diameter were less than $40\,cm^{-3}$. To check the repeatability of the measurement, one of the samples of

ammonium sulfate-methylglyoxal was measured three times across a span of 15 days (Figure S16b). The critical diameter D_d ranged between 50.96 and 51.71 nm, which translated to a range in κ parameter values between 0.616 and 0.644. The CCN experimental setup appeared to behave quite consistently, indicating good repeatability of the results.

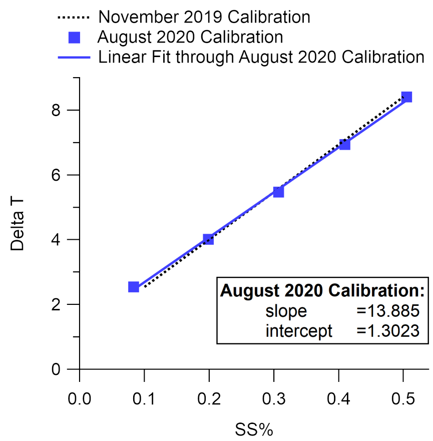

(a) CCNC calibrations throughout the measurements in this work

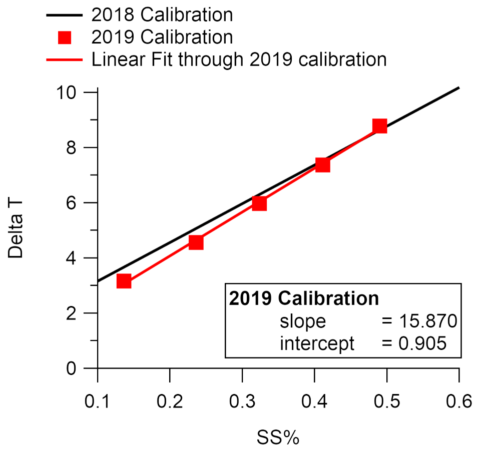

(b) CCNC calibration conducted by Borduas-Dedekind et al.[1], for a similar CCNC instrument

Figure S16: (a) The supersaturation of the CCNC was calibrated following the procedure described by Rose et al.[3]. The supersaturation of the instrument was controlled by a temperature gradient (Delta T). This temperature gradient, monitored by temperature sensors, was converted to a theoretical supersaturation based on CCNC measurements with ammonium sulfate aerosols at six supersaturation conditions (square markers). The slope and intercept of the resulting calibration (green line) was compared to the previous calibration (black line) and corrected accordingly in the CCNC's calibration program. (b) Borduas-Dedekind et al.[1],[5] used a similar CCNC instrument with samples of SRFA which may explain a slight discrepency between the two measurements of the same SRFA solution, yet it was calibrated using the same calibration procedure, yielding a similar calibration curve.

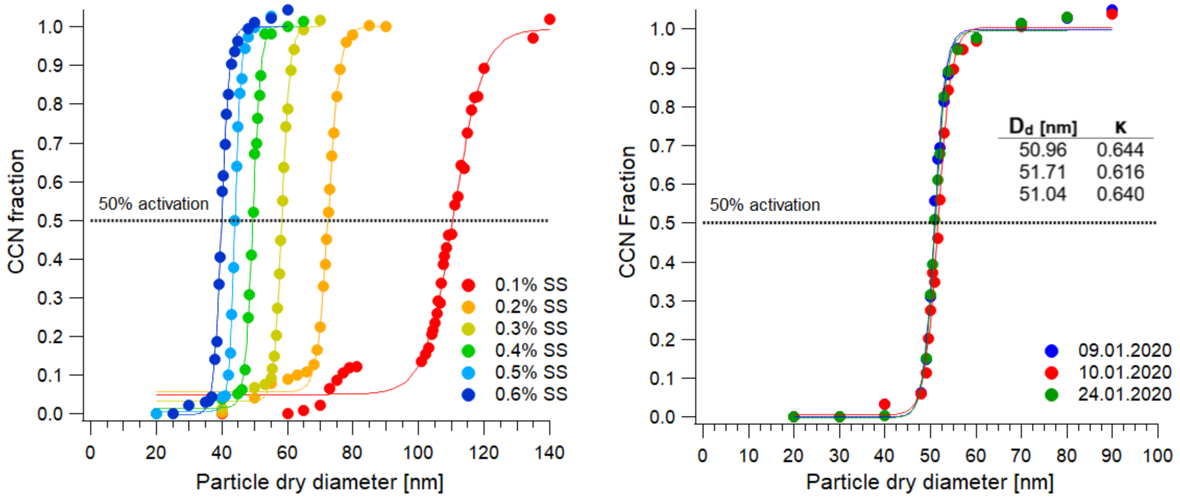

(a) (b)

Figure S17: (a) Activated CCN fraction as a function of particle diameter for an ammonium sulfate solution (65 mg of ammonium sulfate in 100 mL of Milli-Q water) at supersaturations (SS) between 0.1% and 0.6%. A sigmoidal fit was applied to generate activation curves for each supersaturation, yielding the critical diameter at which 50% of the particles activated into droplets. These data were used to calibrate the CCNC with respect to the effective water vapor supersaturation, following the procedure described by.[3] (b) Triplicate measurements of an ammonium sulfate-methylglyoxal sample conducted on different days up to two weeks apart show good reproducibility with consistent D_d of 50.96-51.71nm and κ values of 0.616-0.644.

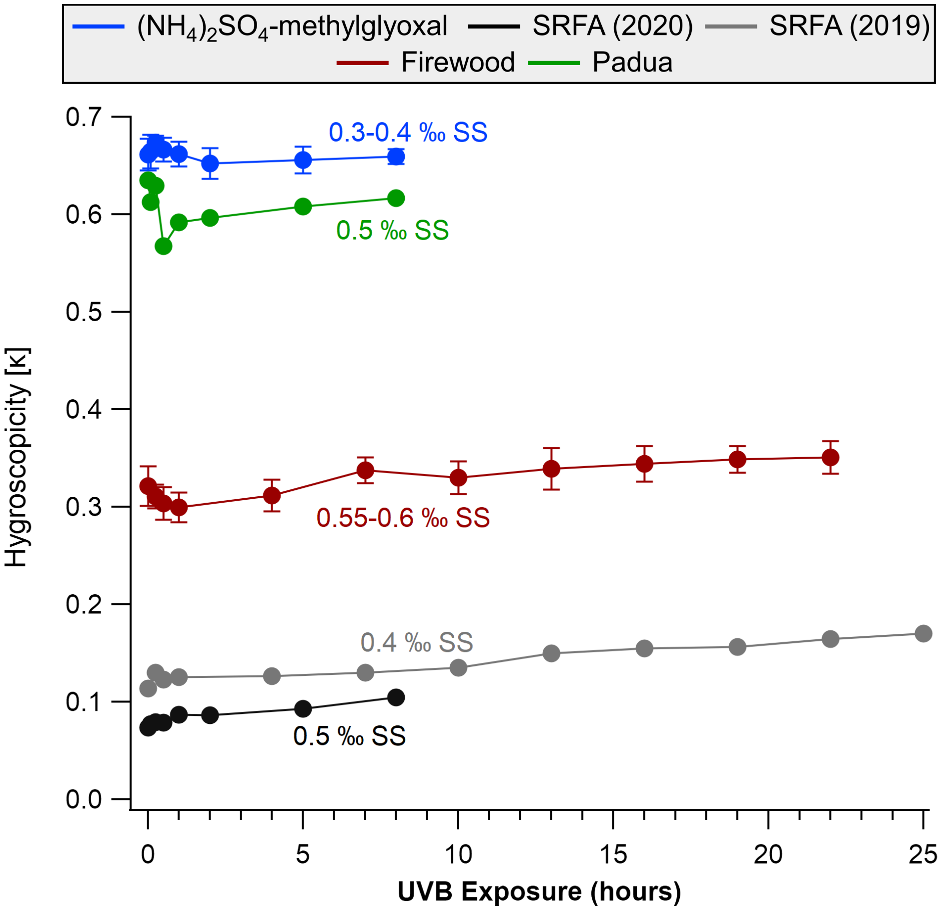

Figure S18: Hygroscopicity represented by kappa values as a function of UVB irradiation for the four types of BrC solutions. The ammonium-sulfate methylglyoxal and firewood solutions were measured in triplicate experiments and the mean and standard deviations are also depicted. The CCNC was operated at 0.30-0.40 SS%, SS%, 0.55-0.60 SS% and at 0.5% for the BrC solution, SRFA, firewood and Padua samples, respectively.

References

(1) Borduas-Dedekind, N.; Ossola, R.; David, R.; Boynton, L.; Weichlinger, V.; Kanji, Z.; McNeill, K. Photomineralization mechanism changes the ability of dissolved organic matter to activate cloud droplets and to nucleate ice crystals. *Atmospheric Chemistry and Physics* **2019**, *19*, 12397–12412.

(2) Appiani, E.; Page, S. E.; McNeill, K. On the Use of Hydroxyl Radical Kinetics to Assess the Number-Average Molecular Weight of Dissolved Organic Matter. *Environmental Science & Technology* **2014**, *48*, 11794–11802.

(3) Rose, D.; Gunthe, S. S.; Mikhailov, E.; Frank, G. P.; Dusek, U.; Andreae, M. O.; Pöschl, U. Calibration and Measurement Uncertainties of a Continuous-Flow Cloud Condensation Nuclei Counter (DMT-CCNC): CCN Activation of Ammonium Sulfate and Sodium Chloride Aerosol Particles in Theory and Experiment. *Atmospheric Chemistry and Physics* **2008**, *8*, 1153–1179.

(4) Topping, D. O.; McFiggans, G. B.; Coe, H. A curved multi-component aerosol hygroscopicity model framework: Part 1 – Inorganic compounds. *Atmospheric Chemistry and Physics* **2005**, *5*, 1205–1222.

(5) Borduas-Dedekind, N.; Nizkorodov, S.; McNeill, K. UVB-irradiated Laboratory-generated Secondary Organic Aerosol Extracts Have Increased Cloud Condensation Nuclei Abilities: Comparison with Dissolved Organic Matter and Implications for the Photomineralization Mechanism. *CHIMIA International Journal for Chemistry* **2020**, *74*, 142–148.
